# RD26 mediates crosstalk between drought and brassinosteroid signalling pathways

**DOI:** 10.1038/ncomms14573

**Published:** 2017-02-24

**Authors:** Huaxun Ye, Sanzhen Liu, Buyun Tang, Jiani Chen, Zhouli Xie, Trevor M. Nolan, Hao Jiang, Hongqing Guo, Hung-Ying Lin, Lei Li, Yanqun Wang, Hongning Tong, Mingcai Zhang, Chengcai Chu, Zhaohu Li, Maneesha Aluru, Srinivas Aluru, Patrick S. Schnable, Yanhai Yin

**Affiliations:** 1 Department of Genetics, Development and Cell Biology, Iowa State University, Ames, Iowa 50011, USA; 2 Department of Agronomy, Iowa State University, Ames, Iowa 50011, USA; 3 Data2Bio LLC, Ames, Iowa 50011-3650, USA; 4 Department of Molecular Biology, Massachusetts General Hospital and Department of Genetics, Harvard Medical School, Boston, Massachusetts 02115, USA; 5 Institute of Genetics, Chinese Academy of Sciences, Beijing 100101, China; 6 State Key Laboratory of Plant Physiology and Biochemistry, Department of Agronomy, College of Agronomy and Biotechnology, China Agricultural University, Beijing 100193, China; 7 School of Biology, Georgia Institute of Technology, Atlanta, Georgia 30332, USA; 8 School of Computational Science and Engineering, Georgia Institute of Technology, Atlanta, Georgia 30332, USA

## Abstract

Brassinosteroids (BRs) regulate plant growth and stress responses via the BES1/BZR1 family of transcription factors, which regulate the expression of thousands of downstream genes. BRs are involved in the response to drought, however the mechanistic understanding of interactions between BR signalling and drought response remains to be established. Here we show that transcription factor RD26 mediates crosstalk between drought and BR signalling. When overexpressed, BES1 target gene *RD26* can inhibit BR-regulated growth. Global gene expression studies suggest that RD26 can act antagonistically to BR to regulate the expression of a subset of BES1-regulated genes, thereby inhibiting BR function. We show that RD26 can interact with BES1 protein and antagonize BES1 transcriptional activity on BR-regulated genes and that BR signalling can also repress expression of *RD26* and its homologues and inhibit drought responses. Our results thus reveal a mechanism coordinating plant growth and drought tolerance.

## Abstract (web summary)

Brassinosteroid (BR) signalling regulates plant development via the BES1/BZR1 family of transcription factors. Here the authors show that BES1 activity can be modified by the drought-responsive RD26 transcription factor providing a molecular basis for the interaction between drought and BR signalling.

Brassinosteroids (BRs) are a group of plant steroid hormones regulating plant growth, development and responses to biotic and abiotic stresses^1^,^2^. Over the past two decades, the main components of the BR signalling pathway have been identified and characterized^3^,^4^,^5^,^6^,^7^,^8^,^9^,^10^,^11^,^12^,^13^,^14^,^15^,^16^,^17^,^18^,^19^,^20^,^21^,^22^. BR signalling leads to the accumulation of BES1/BZR1 (BRI1 EMS SUPPRESSOR 1/BRASSINAZOLE RESISTANT 1) family transcription factors in the nucleus to control the expression of target genes for BR responses^23^,^24^,^25^,^26^,^27^,^28^.

Several studies indicated that treatment of exogenous BRs could enhance the tolerance of plants to drought^1^,^29^,^30^. However, BR-deficient mutants were reported to have an enhanced tolerance to drought^31^,^32^,^33^, suggesting an inhibitory effect of BRs on drought tolerance. These early studies imply complex relationships between BR-regulated growth and drought responses. Several transcription factors, including drought-induced transcription factor RD26 (RESPONSIVE TO DESICCATION 26) and several of its close homologues, have been identified as the direct targets of BES1 and BZR1 (refs 23, 24), suggesting that these proteins may play important roles in interactions between BR and drought pathways.

RD26 belongs to the NAC (No apical meristem, *Arabidopsis* transcription activation factor and cup-shaped cotyledon) family of transcription factors, which are induced by drought, abscisic acid, NaCl and jasmonic acid^34^,^35^,^36^,^37^. Reporter gene expression studies showed that RD26 is expressed constitutively in both shoots and roots upon drought or salt stress treatments^38^,^39^. RD26 and its homologues function to promote drought-responsive gene expression and increase plant drought tolerance^35^. Recent studies showed that RD26 and its homologues, ANAC019 and ANAC055, are involved in plant bacterial pathogenesis, jasmonic acid-mediated defence and thermotolerance^37^,^38^,^39^,^40^,^41^,^42^.

In this study, we confirmed that *RD26* is a target gene of BES1 and negatively regulates the BR signalling pathway. RD26 affects BR-regulated gene expression when overexpressed globally by binding and antagonizing BES1 transcriptional activities. Loss-of-function mutants in the BR signalling pathway had higher drought tolerance, while gain-of-function mutants in the BR pathway exhibited lower drought tolerance compared with wild type (WT). These results suggest that RD26 inhibits BR-regulated plant growth and the BR pathway also negatively regulates drought tolerance, establishing a mechanism for crosstalk between these two important pathways for plant growth and stress responses.

## Results

### RD26 is a negative regulator of the BR signalling pathway

Previous ChIP–chip studies indicated that *RD26* was a target of BES1 and BZR1, and its expression was repressed by BL (brassinolide, the most active BR), BES1 and BZR1 (refs 23, 24). Since BES1 and BZR1 can bind to BRRE to repress gene expression, we examined the *RD26* gene promoter and found a BRRE site at nucleotide position −851 relative to the transcriptional start site. Chromatin immunoprecipitation (ChIP) experiments showed that BES1 binds to the BRRE site *in vivo*, with more binding in *bes1-D* in which BES1 protein accumulates than in WT plants (Supplementary Fig. 1a). *RD26* expression was reduced by BL in WT plants and was repressed in *bes1-D* (Supplementary Fig. 1b). These results confirm that *RD26* is a target of BES1, and its expression is repressed by BL through BES1.

**Figure 1:**
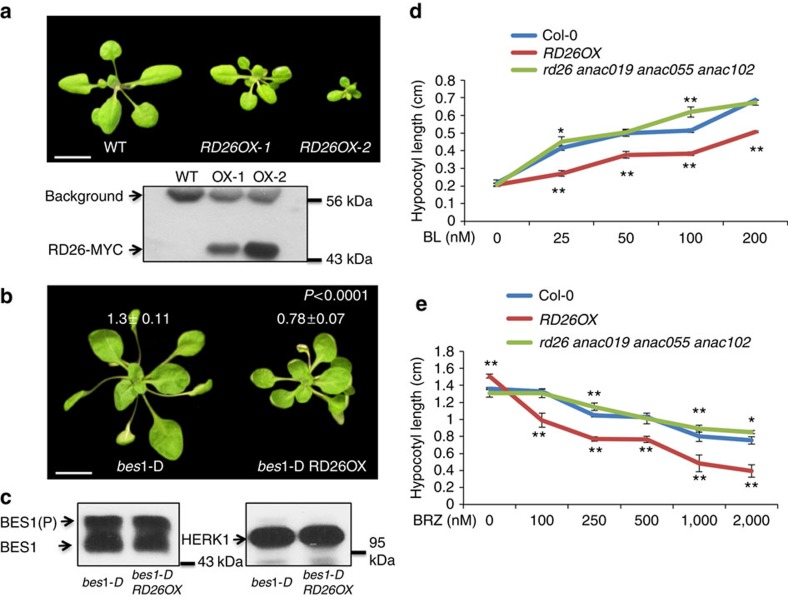
RD26 functions as a negative regulator in the BR signalling pathway. (**a**) The phenotype of 4-week-old *RD26* overexpression (*RD26OX*) plants. The stunted growth phenotype of *RD26OX* plant (upper) is correlated with the protein level of *RD26* transgene (lower panel) examined by western blotting. T3 homozygous plants were used in the experiments and the phenotypes have been stable for many generations after T3. Scale bar, 1 cm. (**b**) *RD26OX* suppressed *bes1-D* phenotype. Four-week-old plants of *bes1-D* and *bes1-D RD26OX* double mutants are shown. Scale bar, 1 cm. The average petiole length of the sixth leaves and s.d. are indicated (*n*=10). (**c**) BES1 protein levels and phosphorylation status did not change in *bes1-D RD26OX* (right lane) compared with *RD26OX* (left lane) as shown by a western blot (left panel). A loading control with HERK1 is also shown (right panel). (**d**) The hypocotyl lengths of 10-day-old light-grown seedlings in the absence or presence of different concentrations of BL. Error bars represent s.d. (*n*=5–10). The experiments were repeated twice with similar results. (**e**) The hypocotyl lengths of 5-day-old dark-grown seedlings in the absence or presence of different concentrations of BRZ. Error bars represent s.d. (*n*=10–15). The experiments were repeated three times with similar results. Significant differences were based on Student's *t*-test (**P<0.01; *P<0.05), which is applied to all other experiments in this study. Also see Supplementary Fig. 1c.

Our previous result indicated that the loss-of-function *rd26* mutant has a small increase in BR response^23^, suggesting that RD26 functions with its homologues to inhibit BR response. To confirm this hypothesis, we generated *RD26* overexpression transgenic lines. *RD26*-overexpressing plants (*RD26OX*) displayed a stunted growth phenotype, the severities of which correspond well with RD26 protein levels (Fig. 1a). Moreover, the *RD26OX* transgenic plants could suppress the phenotype of *bes1-D*, a gain-of-function mutant in the BR pathway (Fig. 1b). Western blotting indicated that BES1 protein levels and phosphorylation status did not change significantly in *bes1-D RD26OX* double mutant (Fig. 1c). These results suggest that RD26 functions downstream of BES1 to inhibit BR-mediated growth.

To confirm that the *RD26OX* phenotype is related to reduced BR response, we determined its response to BL and to the BR biosynthesis inhibitor brassinazole (BRZ), which reduces endogenous BR levels^43^. RD26 overexpression plants have reduced response to BL in hypocotyl elongation assays (Fig. 1d). Likewise, *RD26OX* seedlings had shorter hypocotyls and were more sensitive to BRZ compared with Col-0 WT (Fig. 1e). Several RD26 homologues, *ANAC019*, *ANAC055* and *ANAC102*, are also BES1 and/or BZR1 direct targets, and are repressed by BRs likely functioning redundantly in BR responses^23^,^24^. We generated quadruple mutant of *rd26 anac019 anac055 anac102.* The quadruple mutant has a BR-response phenotype and showed increased response to BL (especially at 100 nM BL) compared with WT (Fig. 1d). The *rd26 anac019 anac055 anac102* quadruple mutant is less sensitive to BRZ, especially at higher concentrations (Fig. 1e and Supplementary Fig.1c). The genetic evidence demonstrates that RD26 and its close homologues play a negative role in the BR signalling pathway.

### RD26 negatively regulates BR-responsive genes

**Figure 2:**
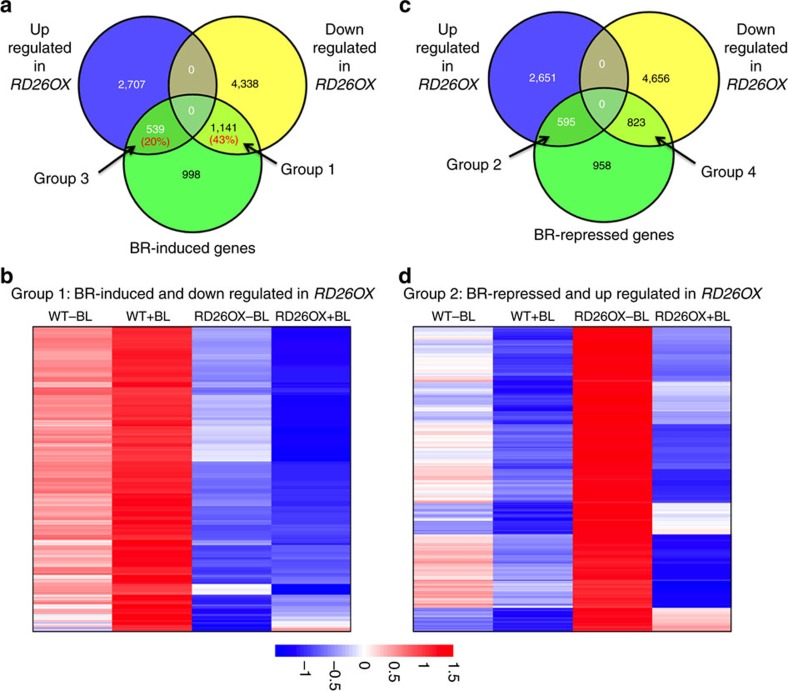
RD26 negatively regulates the expression of some BR-responsive genes. (**a**) Venn diagram shows the overlap between BR-induced genes and *RD26OX*-regulated genes. (**b**) Clustering analysis of Group 1 genes. In all, 1,141 BR-induced genes are downregulated in *RD26OX* plants. (**c**) Venn diagram shows the overlap between BR-repressed genes and genes affected in *RD26OX*. (**d**) Clustering analysis of Group 2 genes. In all, 595 BR-repressed genes are upregulated in *RD26OX* plants.

To determine whether the strong phenotype of *RD26OX* plants is indeed related to BR response, we examined several known BR-induced genes by quantitative PCR (qPCR; Supplementary Fig. 2a). In general, many BR-induced genes we tested are downregulated in *RD26OX*, including genes involved in BR-regulated cell elongation (TCH4 and EXPL2), supporting a role of RD26 in modulating BR-regulated gene expression and plant growth. To more fully understand how RD26 negatively regulates BR responses, we performed global gene expression studies with *RD26* mutants in the absence or presence of BRs by high-throughput RNA-sequencing (RNA-seq). We used 4-week-old adult plants for gene expression studies because *RD26OX* plants display the most obvious growth phenotype at this stage. In WT, 2,678 genes were induced and 2,376 genes were repressed by BL, among ∼22,000 genes analysed (Fig. 2 and Supplementary Data 1 and 2), as we previously reported^44^. The BR-regulated genes from our RNA-seq analysis in adult plants have significant overlaps (∼43%) with previous microarray analyses of BR-regulated genes in either seedlings or adult plants (Supplementary Data 3 and 4 and Supplementary Fig. 2b)^24^,^45^,^46^,^47^,^48^,^49^. Consistent with the strong phenotype of *RD26OX* plants, 3,246 genes are upregulated and 5,479 genes are downregulated in the transgenic plants, respectively (Fig. 2 and Supplementary Data 5 and 6).

To explore how RD26 affects BR-regulated gene expression, we examined the overlaps between BR-regulated genes and genes affected in *RD26OX* plants by performing clustering analysis with specific gene groups. RD26 modulates BR-responsive genes in complex ways (Fig. 2 and Supplementary Fig. 3). Consistent with the negative role of RD26 in BR response, 43% (1,141, Group 1) of BR-induced genes were downregulated in *RD26OX* plants and their induction by BRs was reduced, but not abolished (Fig. 2a,b). In contrast, only 20% (539, Group 3) of BR-induced genes were upregulated in *RD26OX* (Fig. 2a and Supplementary Fig. 3a). These results suggest that RD26 negatively modulates a significant portion of BR-induced genes.

On the other hand, among 2,376 BR-repressed genes, 595 (25%, Group 2) were upregulated and 823 (35%, Group 4) were downregulated in *RD26OX* plants (Fig. 2c,d and Supplementary Fig. 3b). While Group 3 and Group 4 genes suggest a positive role for RD26 in BR response (that is, BR-induced genes are upregulated and BR-repressed genes are downregulated in *RD26OX*), Group 1 and Group 2 genes demonstrated a negative role of RD26 in BR response (BR-induced genes are downregulated and BR-repressed genes are upregulated in *RD26OX*). In this study, we focus on the Group 1 and Group 2 genes to determine the mechanisms by which RD26 negatively regulates BR responses.

Consistent with the relatively weak BR-response phenotype of the *rd26 anac019 anac055 anac102* mutant, only 405 genes are upregulated and 378 are downregulated in *rd26 anac019 anac055 anac102* quadruple mutant (Supplementary Fig. 4 and Supplementary Data 7 and 8). We further compared BR-regulated genes and genes affected in *RD26OX* and the *rd26 anac019 anac055 anac102* mutant (Supplementary Fig. 5a,b). Four subgroups are subjected to further clustering analysis: BR-induced genes that are downregulated in *RD26OX* and upregulated in the quadruple mutant (36, Supplementary Fig. 5c); BR-induced genes that are upregulated in *RD26OX* and downregulated in the quadruple mutant (15, Supplementary Fig. 5e); BR-repressed genes that are upregulated in *RD26OX* and downregulated in the quadruple mutant (44, Supplementary Fig. 5d); and BR-repressed genes that are downregulated in *RD26OX* and upregulated in the quadruple mutant (19, Supplementary Fig. 5f). Most of these genes are affected in opposite ways in the *rd26 anac019 anac055 anac102* mutant and *RD26OX*. These results support the conclusion that RD26 and its homologues function in a complex way to modulate BR-regulated gene expression.

### RD26 and BES1 differentially control BR-regulated genes

**Figure 3:**
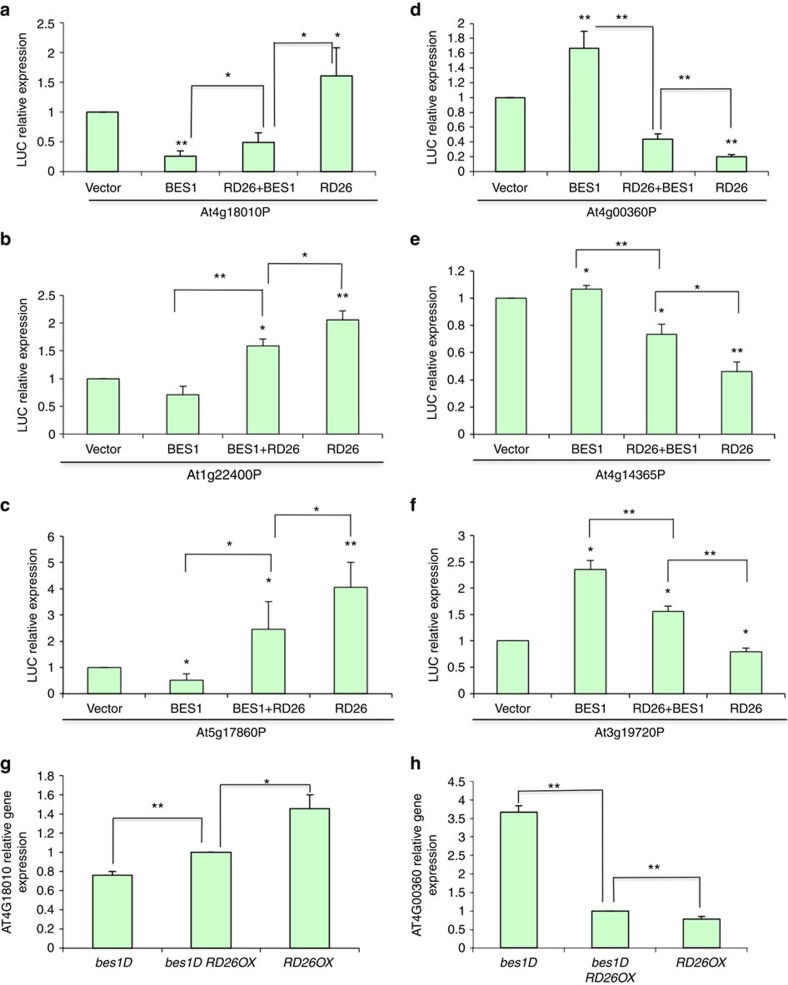
RD26 inhibits BES1 transcriptional activity. (**a**–**f**) Transient gene expression assays were performed in tobacco leaves with *indicated gene promoters-LUC* reporter genes co-transfected with *BES1* and/or *RD26* via Agrobacterium. The relative expression levels of luciferase were normalized with total protein. Error bars represent s.d. from three to five biological replicates. (**g**,**h**) The expression of *At4g00360* and *At4g18010* were examined in *bes1-D*, *bes1-D RD26OX* double mutant and *RD26OX* by qPCR. Error bars represent s.d. from three biological replicates. Significant differences were based on Student's *t*-test (**P<0.01; *P<0.05).

Previous studies indicated that both BES1 and BZR1 can bind to the BRRE site or E-boxes to inhibit or activate gene expression, respectively^23^,^24^. We examined the Group 1 and Group 2 gene promoters and found that BRRE elements are especially enriched in Group 2 gene promoters (Supplementary Fig. 6a and Supplementary Table 1) and E-boxes (CANNTG, especially a specific E-box CATGTG in BR-induced gene promoters^28^) are enriched in Group 1 gene promoters (Supplementary Fig. 6b,c and Supplementary Table 1), within 500 base pairs (bp) relative to the transcriptional start sites. The differential enrichments within −500 bp promoter regions are significant as most BES1- and BZR1-binding sites are located in the region as revealed by genome-wide ChIP–chip studies^23^,^24^. We selected several gene promoters from Group 1 and Group 2 and fused with *luciferase* (*LUC*) gene to generate reporter constructs. *BES1*, *RD26* or *BES1* plus *RD26* were co-expressed with the reporter constructs and the reporter gene expression was determined. While BES1 repressed and RD26 activated the expression of Group 2 genes, the reporter gene expression level was in between when *BES1* and *RD26* were co-expressed (Fig. 3a–c). In contrast, BES1 activated and RD26 repressed Group 1 reporter genes, and the expression level fell in the middle when *RD26* and *BES1* were co-expressed (Fig. 3d–f). These results indicated that RD26 acts to antagonize BES1 actions on these BR-regulated genes.

To reveal the mechanisms by which RD26 inhibits the large number of BR-induced genes (Group 1, Fig. 2b) and upregulates many BR-repressed genes (Group 2, Fig. 2d), we chose one gene representative of each group for further mechanistic studies. A BR-repressed gene, *At4g18010*, was chosen to represent Group 2 genes because it is upregulated in *RD26OX* and its promoter contains a BRRE site at −405 bp relative to the transcription start site (Supplementary Fig. 7a). Likewise, A BR-induced gene *At4g00360* was chosen to represent Group 1 genes as its promoter contains a well-established BES1-binding site, CATGTG E-box, at nucleotide −470 (Supplementary Fig. 7b).

To confirm the antagonistic effect of RD26 on BES1-mediated gene expression observed by LUC reporter gene assays, we examined the expression of these two genes in *bes1-D*, *RD26OX* and *bes1-D RD26OX* plants, in which BES1, RD26 or both are increased. As shown in Fig. 3g, the expression of *At4g18010* was downregulated in *bes1-D* and upregulated in *RD26OX*, but the expression level was in between in *bes1-D RD26OX* double mutant. In contrast, the expression of *At4g00360* was much higher in *bes1-D* compared with *bes1-D RD26OX*, while its expression was significantly repressed in *RD26OX* (Fig. 3h).

### RD26 and BES1 bind to promoters simultaneously

Previous DNA-binding experiments showed that NAC transcription factors including RD26 (ANAC072) and ANC019 could bind to DNA sequences with two motifs—CATGT(G) and a CACG core spaced by varying numbers of nucleotides^35^,^41^,^42^. The NAC-binding sites are very similar to E-box (CANNTG) or conserved core sequence of the BRRE site (CGTGT/CG), well-established binding sites for BES1/BZR1 (refs 23, 24). These results suggest that RD26 and BES1 could potentially bind to the same site to modulate BR-regulated gene expression.

**Figure 4:**
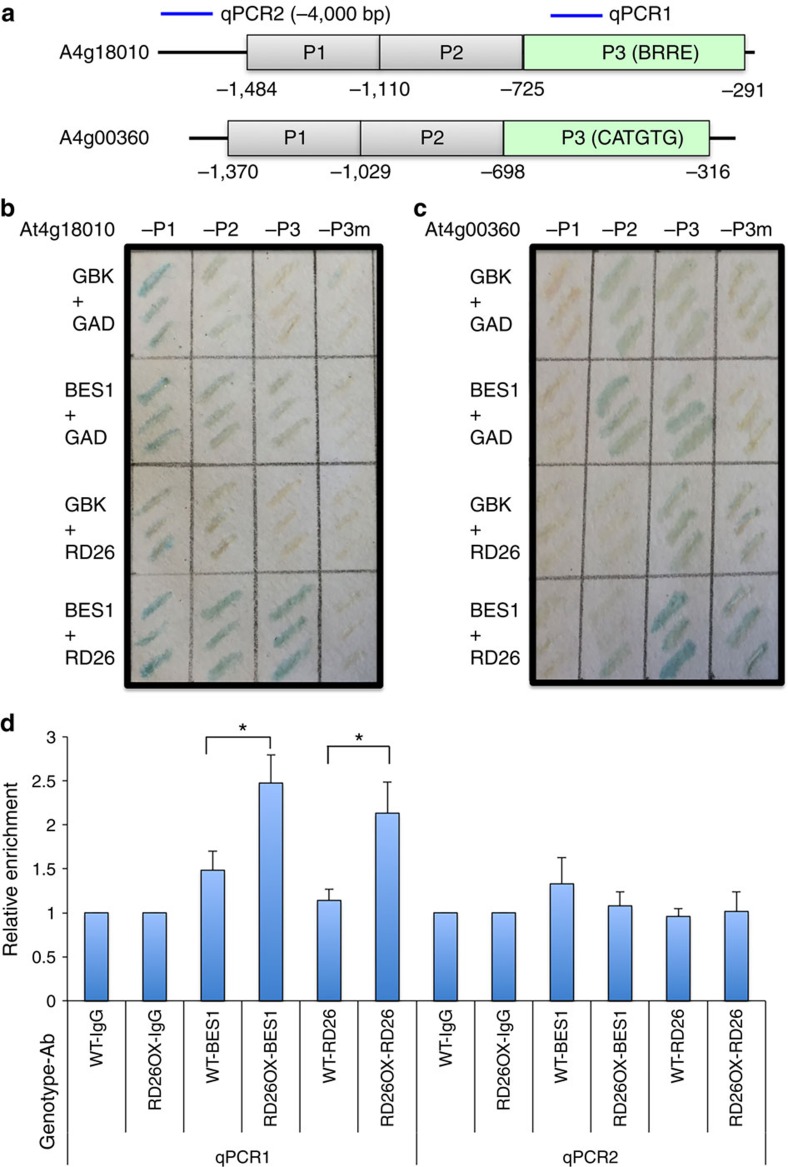
BES1 and RD26 act together through E-box or BRRE sites in target gene promoters. (**a**) At4g18010 and At4g00360 promoters were divided into three fragments based on known BRRE site and CATGTG E-box present in P3 fragments. Mutant P3 fragments (P3m) in which BRRE and CATGTG E-box was mutated (see Fig. 5a) were also generated. Each fragment was cloned into in yeast one-hybrid vector pLacZi (Clontech Inc.) and integrated into yeast strain YM4271. (**b**) BES1 (in pGBKT7, TRP marker), RD26 (in pGADT7, LEU marker), BES1+RD26 were transformed into yeast reporter strains described in **a** with control plasmids and selected in media lacking LEU and TRP. The yeast colonies were grown on filter paper for LacZ assays. BES1 and RD26 seem to be able to function through At4g18010-P2, although there are no known BES1 or RD26-binding sites in this fragment (Supplementary Fig. 7). (**c**) At4g00360-P3 reporter was activated when both BES1 and RD26 are expressed in yeast, but not activated when either BES1 or RD26 are expressed. (**d**) BES1 binding to At4g18010 promoter is enhanced in *RD26OX* plants as revealed by ChIP assays. WT and *RD26-MYC* overexpression plants (*RD26OX*) were used to prepare chromatin and ChIP with antibodies (Ab) against BES1, RD26 or IgG control. The ChIP products were used to detect At4g18010 using primers for qPCR1 (within P3 fragment, see **a**) and qPCR2 (about −4,000 bp upstream of the transcriptional start site). Error bars represent s.e.m. from four biological replicates. The significance of enrichment was determined by Student's *t*-test (*P<0.05).

**Figure 5:**
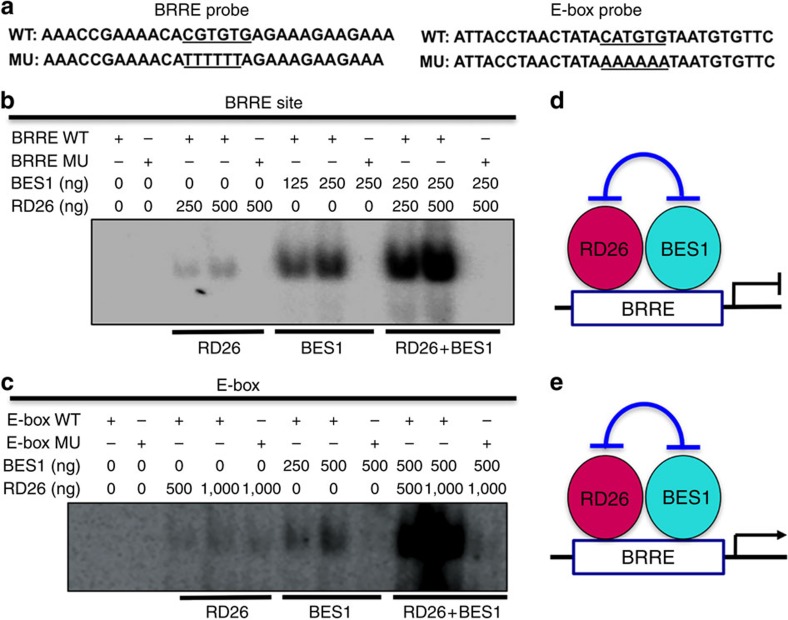
BES1 and RD26 synergistically bind to BRRE or E-box sequence of BR-responsive genes. (**a**) DNA sequences used for binding assays. WT and mutant (MU) forms of BRRE- and E-box-containing probes are shown. (**b**) BES1 and RD26 bind to BRRE element. The DNA sequences containing WT or mutated form of BRRE (CGTGTG) from At4g18010 used as probes for DNA binding are shown. WT or MU probes were labelled with ^32^P-ATP and used in binding reactions with indicated amount (ng) of recombinant proteins. (**c**) BES1 and RD26 bind to E-box. The DNA sequences containing WT or mutated form of E-box (CATGTG) from At4g00360 used as probes for DNA binding are shown. (**d**) A model of RD26 and BES1 binding to BRRE shows that RD26 and BES1 inhibit each other's transcriptional activities. (**e**) A model of RD26 and BES1 binding to E-box shows that that RD26 and BES1 inhibit each other's transcriptional activities.

We first used yeast one-hybrid assays to test whether BES1 and RD26 can target to the same promoter fragments (Fig. 4). We fused several fragments of the At4g18010 promoter (-P1, -P2 and -P3, with BRRE located in P3) and At4g00360 promoter (-P1, -P2 and -P3, with CACGTG E-box located in P3) to pLacZi reporter (Clontech Inc.) and integrated them into the yeast genome (Fig. 4a). Mutants were also generated in which At4g18010-P3 BRRE and At4g00360-P3 E-box were mutated to unrelated sequences (see Fig. 5a). BES1 (with pGBKT7 vector), RD26 (with pGADT7 vector) or both BES1 and RD26 were expressed in each of the reporter yeast strain and the LacZ expression was determined. As shown in Fig. 4b, while neither BES1 nor RD26 significantly changed the gene expression from At4g18010-P3, co-expression of BES1 and RD26 activated the reporter gene expression. It is worth noting that the fusion of the GAL4 activation domain in pGADT7 to RD26 apparently changed RD26 property in yeast to become an activator in combination with BES1 (compared with the result from plants in Fig. 3), which is necessary to detect BES1/RD26 interaction in yeast. Moreover, mutation of the BRRE in At4g18010-P3 completely abolished the activation (Fig. 4b). The results demonstrated that BES1 and RD26 act through the BRRE site in the At4g18010-P3 promoter fragment. Similarly, co-expression of BES1 and RD26 activated At4g00360-P3 reporter, which is much reduced when the CATGTG E-box is mutated, indicating that BES1 and RD26 act through the CATGTG E-box in At4g00360-P3 (Fig. 4c) to regulate gene expression.

We also performed ChIP assays with WT and *RD26OX* transgenic plants, with BES1 antibody^23^ or RD26 antibodies we generated (Supplementary Fig. 8). While BES1 itself binds to the At4g18010 promoter (P3) in WT plants, such binding is enhanced in *RD26OX* plants (Fig. 4d, columns 3 and 4), suggesting that BES1 and RD26 together enhance binding to the promoter region. Consistent with the result that RD26 antibody detects RD26 in *RD26OX* but not in WT plants (Supplementary Fig. 8a,b), RD26 binding to the At4g18010 promoter (P3) in *RD26OX* was strongly apparent but barely detectable in WT (Fig. 4d, columns 5 and 6). In contrast, such cooperative binding is not detected in the more upstream promoter region (Fig. 4d, columns 9–12).

To confirm that BES1 and RD26 can bind to the same promoter regions at the same time, we also performed ChIP–reChIP with chromatin prepared from *RD26OX, rd26 anac019 anac055 anac102 (rdQ) or BES1 RNAi* plants in which the BES1 level is reduced^27^ (Supplementary Fig. 9). When the first ChIP was performed with anti-BES1 antibody and eluted chromatin samples were then immunoprecipitated with anti-RD26 or IgG control, significant enrichment of BES1/RD26 binding was detected in *RD26OX* plants, which is clearly reduced in *rdQ* mutant, and moderately reduced in *BES1RNAi* plants with two pairs of independent qPCR primers (Supplementary Fig. 9a,b). Similar results were obtained when the first ChIP was performed with anti-RD26 antibody and reChIP with anti-BES1 (Supplementary Fig. 9c). These results suggest that BES1 and RD26 can simultaneously bind to the At4g18010 gene promoter *in vivo*.

To further reveal the biochemical mechanisms by which RD26 antagonizes BES1 actions, electrophoretic mobility shift assay (EMSA) experiments were performed with recombinant BES1 and RD26 proteins using DNA probes containing BRRE (from At4g18010) or CATGTG E-box (from At4g00360; Fig. 5a and Supplementary Fig. 10). While BES1 binds to BRRE (CGTGTG) from At4g18010 quite strongly, RD26 binds to the probe more weakly; moreover, both bindings were abolished with mutant probe in which BRRE is mutated (Fig. 5b). Interestingly, BES1 and RD26 together can bind to the BRRE probe more strongly, and the binding is also abolished when the BRRE site is mutated (Fig. 5b). Similar results were obtained with probe containing CATGTG E-box from At4g00360. While RD26 and BES1 can each bind to E-box site separately, RD26 and BES1 synergistically bind to WT but not mutated E-box probe (Fig. 5c). Since BES1 (335 aa) and RD26 (298 aa) are similar in predicted protein sizes, the strong bands when both proteins are present more likely represent heterodimer of the two proteins, while each of them likely bind to the probe as homodimer. Taken together, the DNA binding and gene expression results suggest that RD26 interacts with BES1 on BRRE site to inhibit BES1's repression function (Fig. 5d) and on E-box to inhibit BES1's activation function (Fig. 5e).

**Figure 6:**
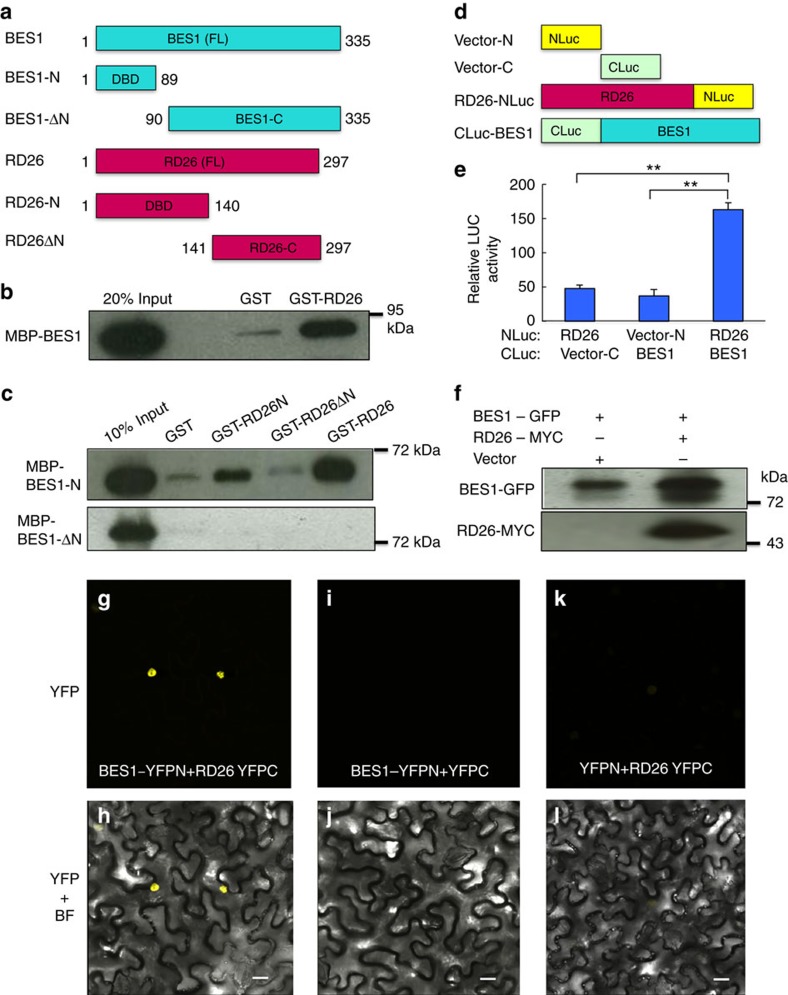
RD26 interacts with BES1 *in vitro* and *in vivo*. (**a**) Schematic representation of BES1 and RD26 proteins. Full-length (FL) or domains involved DNA binding/dimerization for BES1 (aa 1–89) or RD26 (aa 1–140) are shown. (**b**) BES1 interacts with RD26 in glutathione *S*-transferase (GST) pull-down assays. GST-RD26, but not GST, pulled down BES1. MBP-BES1 was detected by ant-MBP antibody. MBP-BES1 (20%) input is shown. (**c**) The DNA binding and dimerization domains of BES1 (1–89) and RD26 (1–140) interacts with each other. (**d**) Schematic representation of constructs used for split-LUC assays. Amino or carboxyl parts of Luciferase (NLuc and CLuc) were fused with RD26 or BES1, respectively. (**e**) RD26 and BES1 interact with each other *in vivo*. RD26-NLuc and CLuc-BES1 as well as indicated controls were co-expressed in tobacco leaves and Luc activities were measured and normalized against total protein. The averages and s.d. of relative luciferase activities were derived from six independent biological replicates. All the experiments were repeated three times with similar results. (**f**) BES1 interacts with BES1 through co-immunoprecipitation assay. BES1-GFP and RD26-MYC were co-expressed in tobacco leaves and protein extract was immunoprecipitated with anti-GFP antibody and detected with anti-BES1 (top panel) or anti-MYC (bottom panel) antibodies. (**g**–**l**) BES1 interacts with RD26 in BiFC assays. Co-expression of 35S:BES1-YFPN with 35S:RD26-YFPC in tobacco leaves led to reconstitution of YFP signal in the nucleus. No positive signal was observed in control samples co-expressing 35S:BES1-YFPN and 35S:YFPC or 35S:YFPN and 35S:RD26-YFPC. For each panel YFP as well as YFP and bright field (BF) merged images (YFP + BF) from confocal microscopy are shown. Scale bars, 20 μm. The experiments were repeated three times with similar results. Significant differences were based on Student's t-test (**P<0.01).

The yeast one-hybrid and DNA-binding experiments described above suggest that BES1 and RD26 may be able to interact with each other. To test this hypothesis, we expressed full-length or truncated BES1 with MBP, and RD26 and truncations with Glutathione *S*-transferase tag, respectively (Fig. 6a). Glutathione *S*-transferase pull-down assays indicated that full-length RD26 could interact with full-length BES1 protein (Fig. 6b). The domains involved in DNA binding/dimerization of BES1 (aa 1–89) and RD26 (aa 1–140) are sufficient for the interaction (Fig. 6c). Split Luciferase (Luc) assay was used to test whether RD26 and BES1 interact in plants^50^. RD26 was fused with the amino part of Luc (NLuc) and BES1 was fused with carboxyl-part of Luc (CLuc), respectively (Fig. 6d). Co-expression of RD26-NLuc and CLuc-BES1 in tobacco leaves led to increased Luc activity, while co-expression of controls (RD26-NLuc with CLuc or CLuc-BES1 with NLuc) only produced background-level activities (Fig. 6e).

We further confirmed that BES1 and RD26 interaction *in vivo* by co-immunoprecipitation and by BiFC experiments. GFP antibody (tagged to BES1) can specifically pull down RD26-MYC co-expressed in tobacco leaves (Fig. 6f). In BiFC assays, co-expression of BES1-YFPN and RD26-YFPC lead to reconstitution of yellow fluorescence protein (YFP) signal in the nucleus (Fig. 6g,h), but YFP signals were not observed in BES1-YFPN/YFPC or YFPN/RD26-YFPC controls (Fig. 6i–l). Taken together, these results indicated that BES1 and RD26 can interact with each other through corresponding DNA-binding/dimerization domains and inhibit each other's functions on Group 1 and Group 2 genes.

### The BR signalling pathway inhibits drought response

Since BRs function through BES1/BZR1 to repress the expression of RD26 and its homologues, we tested whether the BR pathway affects plant drought response. Previous data showed that the expression of RD26 was induced by drought^29^,^30^,^35^,^38^. Drought induces 2,503 and represses 2,862 genes (combination of 2- and 3-day drought treatment data, Supplementary Data 9 and 10)^51^. Analysis of gene expression affected in *RD26OX* and drought-regulated genes revealed that RD26 upregulated 38% (963) of drought-induced genes, but only 12% (346) of drought-repressed genes; similarly, RD26 downregulated 45% (1299) of drought-repressed genes, but only 19% (488) of drought-induced genes (Supplementary Fig. 11). The results suggest that RD26 plays a major role in plant drought responses.

**Figure 7:**
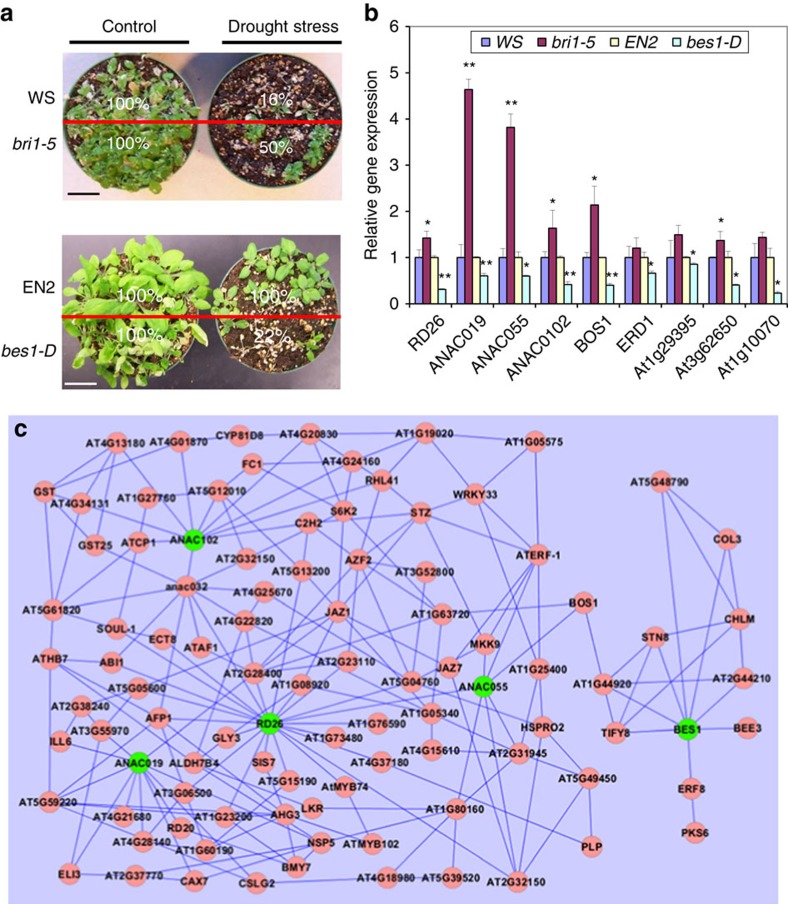
The BR signalling pathway inhibits drought response. (**a**) BR loss-of-function mutant plants (*bri1-5*) have increased and gain-of-function BR mutants (*bes1-D*) have decreased drought tolerance. Survival rates of WS (WT), *bri1-5* mutant, *EN2* (WT) and *bes1-D* mutant plants after withholding water for 14–20 days (drought stress) and rehydration for 7 days (rehydration). The survival rate is indicated in the picture. Scale bars, 3 cm. This experiment was repeated three times with similar results. (**b**) Drought-responsive genes are upregulated in *bri1-5* and downregulated in *bes1-D* mutants. The expression levels of drought-induced genes were examined by qPCR using RNA prepared from *bri1* and *bes1-D* mutants. Error bars indicate s.d. (*n*=3). The difference was significant based on Student's *t*-test (*P<0.05, **P<0.01). (**c**) RD26-BES1 GRN. A 103-gene subnetwork extracted from the *Arabidopsis* whole-genome network^55^ using the subnetwork analysis tool, GeNA. Seed genes (ANAC019, RD26, ANAC055, ANAC102 and BES1) are shown in green. The network topology is displayed using Cytoscape.

We also compared BR-regulated genes and drought-regulated genes and found that ∼38% of BR-regulated genes are modulated by drought (Supplementary Fig. 12). If BR signalling indeed inhibits drought response, we expect that loss-of-function BR mutants have increased, and gain-of-function mutants have decreased, drought tolerance. BR loss-of-function mutant, *bri1-5*, a weak BR receptor mutant^52^, was exposed to drought stress. After drought stress and recovery, 50% of *bri1-5* mutant plants survived, compared with 16% for WT (Fig. 7a, top panel). On the other hand, a gain-of-function mutant in the BR pathway, *bes1-D*, showed less drought tolerance. Only 22% of *bes1-D* mutants survived, but all of WT controls survived in the drought stress experiment (Fig. 7a, bottom panel). The drought response phenotypes were also confirmed in *bes1-D* in Col-0 background^53^ with the same trend (Supplementary Fig. 13).

To test our hypothesis that the BR signalling pathway inhibits drought response by repressing *RD26* and its homologues, the expression of several drought-induced or drought-related genes were examined in *bri1-5* mutant and *bes1-D* mutant. Transgenic plants overexpressing *RD26/ANAC072, ANAC019 or ANAC055* could enhance the tolerance to drought stress, suggesting that RD26 and its homologues ANAC019 and ANAC055 are involved in drought response^19^. Reverse transcriptase qPCR (RT–qPCR) results showed that the expression of all three genes plus *ANAC102* are increased in *bri1-5* mutant and decreased in *bes1-D* mutant (Fig. 7b). We also examined five other genes involved in drought tolerance^54^. All five genes are upregulated in *bri1-5* and downregulated in *bes1-D* (Fig. 7b). The results demonstrated that drought response genes are constitutively expressed in loss-of-function BR mutants and repressed in gain-of-function BR mutants, confirming that the BR signalling pathway inhibits drought response, likely by repressing the expression of *RD26* and its homologues.

We examined the double-mutant *bes1-D RD26OX* and found that RD26 overexpression can clearly rescue the *bes1-D* phenotype in drought response (Supplementary Fig. 14a). Consistent with the facts that RD26OX suppress *bes1-D* phenotypes, several *bes1-D*-induced genes are downregulated in *RD26OX* plants (Supplementary Fig. 14b). The expression of these genes is also reduced in *bes1-D RD26OX* double-mutant compared with *bes1-D* (Supplementary Fig. 14b). The gene expression studies support the idea that *RD26* suppresses *bes1-D* phenotypes.

To further understand the relationships among BES1 and RD26/its close homologues, we constructed a Gene Regulatory Network (GRN) based on gene expression correlations using *BES1, RD26, ANAC019, ANAC055* and *ANAC102* as seed genes^55^. The GRN showed that *RD26* and three of its close homologues have extensive expression correlations, directly or through other genes (Fig. 7c). Interestingly, *BES1* has relatively fewer connections to other genes; in addition, the ‘RD26/homologue cluster' and ‘BES1 cluster' are connected through only one gene, *BOS1*, which was implicated in plant responses to drought, high salinity and fungal pathogens^54^,^56^.

To validate the GRN, we compared the genes in the network with genes affected in *RD26OX*, as well as drought- and BR-regulated genes (Supplementary Fig. 15). Interestingly, 82% of the 103 genes in the GRN are affected in *RD26OX*, although only about one-third of total detected genes are affected in *RD26 OX* plants. Similarly, 72 and 52% of the genes in the GRN are either regulated by drought or BRs, despite the fact that only about one-fourth of total genes are regulated by drought or BRs. The computationally generated GRN and its validation by RNA-seq data support the conclusions that (1): there are close interactions between the BES1-mediated BR pathway and the drought pathway represented by RD26 and its homologues; (2) although the interactions between BES1 and RD26 can happen at a transcriptional level (that is, through BOS1), post-transcriptional regulations such as protein–protein interaction between RD26 and BES1 likely play a major role.

## Discussion

**Figure 8:**
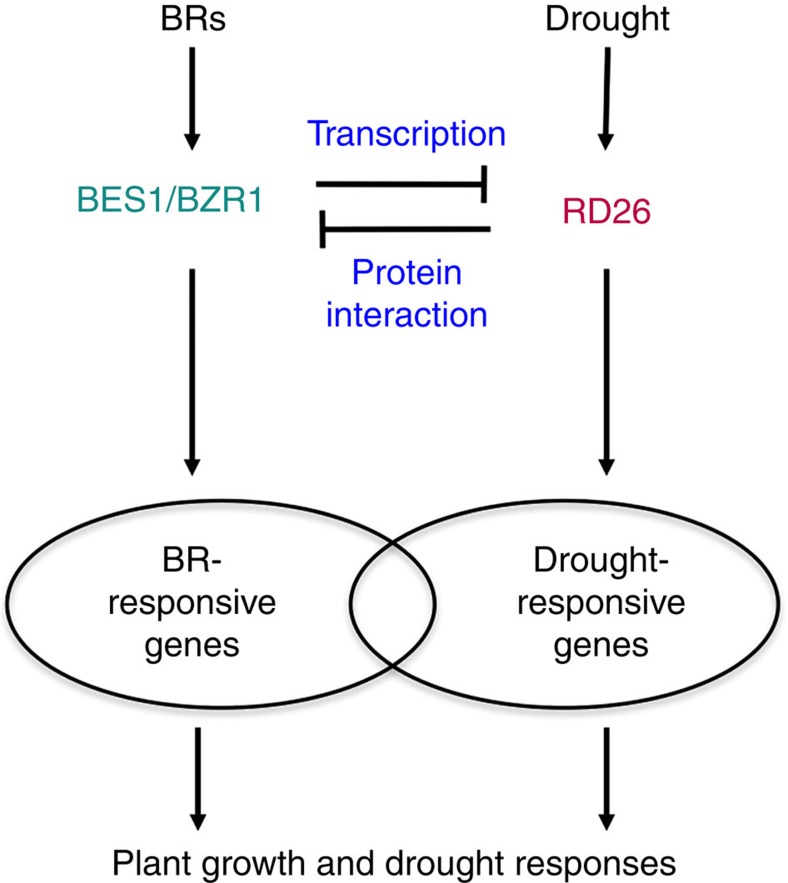
A model of crosstalk between BR and drought response pathways. Drought stress induces the expression of *RD26* to mediate the response of plants to drought. Upon the increased expression, RD26 not only inhibits the expression of BES1 at the mRNA level, but also binds to E-box and BRRE site to inhibit BES1's functions in mediating BR-regulated gene expression (Group I and II genes), which results in the inhibition of BR-regulated growth. On the other hand, BR signalling represses the expression of *RD26* through BES1 and also directly inhibits the expression of other drought-related genes to inhibit drought response.

In this study, we found that the drought-responsive transcription factor *RD26* is a target of BES1 and functions to inhibit BR responses. Gene expression studies revealed that RD26 and BES1 act antagonistically in the regulation of many BR-regulated genes. The antagonistic interactions happen at multiple levels. While BES1/BZR1 functions to repress the expression of *RD26* at a transcription level, the RD26 protein interacts with BES1 and inhibits its transcriptional activity. Our results thus establish a molecular link and mechanism of interaction between BR and drought response pathways (Fig. 8).

Our genetic, genomic, molecular and biochemical results demonstrated that RD26 functions to inhibit the BR pathway (Fig. 8). RD26 is induced by drought, promotes drought-regulated gene expression and confers drought tolerance when overexpressed^35^,^36^. Our genetic studies demonstrate that RD26 is a negative regulator of the BR pathway as overexpression of *RD26* leads to reduced plant growth and BR response and knockout of *RD26* and three of its homologues lead to increased BR response. The relatively weak growth phenotype of *rd26 anac019 anac055 anac102* mutant may be explained by additional family members, which possibly function redundantly in the inhibition of BR response. The fact that a smaller number of genes affected in *rd26 anac019 anac055 anac102* mutant compared with RD26OX transgenic plants is consistent this hypothesis. RD26 and its homologues appear to function as part of a highly redundant and complex network to confer drought tolerance and to inhibit plant growth during drought stress.

Global gene expression studies revealed that RD26 functions to modulate BR-responsive gene expression in a complex manner, that is, RD26 can either activate or repress both BR-induced and BR-repressed genes. However, a large number of BR-induced genes (1,141 or 43% of BR-induced genes identified in this study) are significantly downregulated in *RD26 OX* (Group 1, Fig. 2). Our molecular and biochemical studies suggest that RD26 affects Group 1 gene expression by binding to the BES1 target site (E-box) and neutralizing BES1 activation activity, potentially by forming an inactive heterodimer (Figs 3, 4, 5). Likewise, 595 (or 25%) BR-repressed genes are upregulated in *RD26OX*, suggesting that BR and RD26 have opposite function on these genes (Group 2, Fig. 2). Indeed, the molecular and biochemical evidence suggests that, while BES1 binds to BRRE to repress gene expression, RD26 can antagonize BES1-mediated gene repression (Fig. 3). We also provided evidence that BES1 and RD26 protein can interact with each other *in vitro* and *in vivo* (Fig. 6). While many protein–protein interactions between transcription factors synergistically activate or repress transcription, our results suggest that BES1 and RD26 interact and antagonize each other's transcriptional activities on Group 1 and Group 2 gene promoters. Our findings thus reveal a previously unknown mechanism that two signalling pathways converge on the same promoter element through two interacting transcription factors to coordinate plant growth and stress responses. Consistent with our conclusion, recent ChIP-seq studies showed that RD26 target gene promoters under abscisic acid treatment are enriched in G-box sequence (CACGTG, a specialized E-box)^57^, very similar to BES1 target sites derived from ChIP–chip study^23^.

We also observed an inhibitory effect of the BR pathway on drought response as a loss-of-function BR mutant is resistant to drought and a gain-of-function mutant of the BR pathway had compromised drought response. The transcriptional repression of RD26 and its homologue genes by BRs likely play a major role in the observed inhibition of drought response by the BR pathway as the expression of *RD26* and its homologues (including *ANAC019, ANAC 055* and *ANAC102*) are significantly increased in *bri1* and decreased in *bes1-D* (Fig. 7b). While we have provided experimental evidence that RD26 antagonizes BES1-mediated gene expression on the BES1 target sites, it remains to be determined whether BES1 inhibits RD26-mediated gene expression on RD26-related drought target genes.

We propose that the antagonistic interaction between BES1 and RD26 likely ensures that plant growth is reduced when plants are under drought stress, under which *RD26* and its homologues are upregulated to inhibit BR-induced growth, thus allowing more resources to deal with the drought stress. On the other hand, under normal growth conditions, i.e., in the absence of drought stress, BR signalling represses the drought pathway by repressing the expression of *RD26* and its homologues.

It is worth noting that RD26 and BES1 do not seem to act antagonistically at all times. For example, 539 BR-induced genes (20%, Group 3) are upregulated and 823 BR-repressed genes (35%, Group 4) are downregulated in *RD26OX* (Supplementary Fig. 3), indicating that RD26 and BES1 act in a similar manner on these two groups of genes. It is possible that RD26 and BES1 target different promoter elements to achieve the positive interactions between RD26 and BES1. It has been suggested that at least under some conditions exogenously applied BR can improve plant drought tolerance^58^. It is possible that under these circumstances the Group 2 and Group 4 genes play more dominant roles than Group 1 and Group 2 genes, which can potentially allow BR to activate some drought-induced genes and repress BR-repressed genes and thus promote drought tolerance. More investigation is needed to better understand the interaction between RD26 and BES1 on Group 3 and Group 4 genes.

In summary, we have identified RD26 as a molecular link that coordinates BR and drought responses. We further found that, while BES1 functions to repress RD26 gene expression, RD26 interacts with BES1 and inhibits BES1 transcriptional activity. This reciprocal inhibitory mechanism not only ensures that BR-induced growth is inhibited under drought conditions, but also prevents unnecessary activation of drought response when plants undergo BR-induced growth.

## Methods

### Plant materials and growth condition

T-DNA insertion mutants, *rd26* (At4g27410, SALK_063576), *anac019* (At1g52890, SALK_096295), *anac055* (At3g15500, SALK_014331) and *anac102* (At5g63790, SALK_030702) were obtained from ABRC (*Arabidopsis* Biological Resource Center). All plants were grown on 1/2MS plates and/or in soil under long day conditions (16 h light/8 h dark) at 22 °C. BRZ and BL response experiments were carried out as previous described^59^. Briefly, seeds were sterilized with 70% ethanol and 0.1% Triton X-100 for 15 min and washed with 100% ethanol three times and dried in filter papers in a sterile hood. The seeds were sprinkled onto half Linsmaier and Skoog medium (Caisson Lab) with 0.7% Phytoblend agar (Caisson Lab) and various concentrations of BRZ (provided by Professor Tadao Asami) or BL (Wako Biochemical). Both BRZ and BL (1 mM stock in dimethylsulphoxide) were added to medium after autoclave and the plates with seeds were placed at 4 °C for 3 days. After exposing to light for 8 h, the plates were wrapped with three layers of aluminium foil and incubated in the dark at 25 °C for 5 days for BRZ response and in the constant light for 7 days for BL response experiments. Hypocotyls were scanned and measured using Image J (https://imagej.nih.gov/ij/). Ten to fifteen hypocotyls were measured, and averages and s.d. were calculated and plotted.

### Plasmid constructs

For MYC-tagged transgenic plants, RD26 genomic sequence including its 5′ UTR was cloned from WT and fused with MYC tag and CaMV 35S promoter into pZP211 vector^60^. For recombinant protein purification, full-length or fragments of *RD26* and *BES1*^48^-coding regions were cloned into the pETMALc-H vector^61^ or pET-42a (Novagen). All primers used in this study are provided in Supplementary Table 2.

### Generation and analysis of transgenic plants

The construct of RD26-MYC driven by 35S promoter was transformed into *Agrobacterium tumefaciens* (stain GV3101), which were used to transform plants by the floral dip method^62^. Transgenic lines were selected on 1/2 MS medium plus 60 g ml^−1^ gentamycin. Transgene expression was analysed by western blotting with 2 μg anti-c-MYC (Sigma, C3956) antibody or HERK1 antibody as control. All the uncropped images for western blots in this study are provided in Supplementary Fig. 16. HERK1 kinase domain^49^ and full-length RD26 recombinant proteins were expressed from pETMALc-H and used to generate polyclonal antibody at the Iowa State University Hybridoma Facility (http://www.biotech.iastate.edu/biotechnology-service-facilities/hybridoma-facility/). About 2 μg of affinity-purified antibody was used in each western blotting in 10 ml.

### Gene expression analysis

For *RD26*, *At4g00360* and *At4g18010* gene expression, total RNA was extracted and purified from 2-week-old plants of different genotypes using the RNeasy Mini Kit (Qiagen). The Mx4000 multiplex Quantitative PCR System (Stratagene) and SYBR GREEN PCR Master Mix (Applied Biosystems) were used in quantitative real-time PCR analysis. For transient expression, promoters for At4g00360 (1,552 bp) and At4g18010 (1,515 bp), At1g22400 (1,922 bp), At5g17860 (1,119 bp), At4g14365 (430 bp) and At3g19720 (411 bp) were cloned and used to drive luciferase reporter gene expression. The BES1-coding region driven by CaMV 35S promoter was cloned into pZP211 vector, while RD26-MYC construct used in transgenic plant generation was also used in transient experiment. Tobacco leaf transient assay^63^ was used to examine the effect of RD26 and BES1 on reporter gene expression either with individual protein or with combination of BES1 and RD26. Equal amount of Agrobacterium cells (measured by OD_600_, adjusted to the same with vector-containing strain) were injected into the leaves of tobacco. The activities of the luciferase were measured in total protein extracts from triplicate samples (collected with a 5 mm leaf puncher with same number of leaf discs in each sample) using Berthold Centro LB960 luminometer with the luciferase assay system following the manufacturer's instruction (Promega). The relative level of luciferase activity was normalized by the total amount protein for each sample.

For global gene expression, total RNA was extracted and purified from 4-week-old plants of different genotypes using the RNeasy Mini Kit (Qiagen). Duplicate RNA samples were subjected to RNA-seq using HiSeq2000 50 bp single-end sequencing in the DNA facility at Iowa State University. Raw RNA-seq reads were subjected to quality-checking and trimming and then aligned to the *Arabidopsis* reference genome (TAIR10) using an intron-aware aligner, Genomic Short-read Nucleotide Alignment Program^64^. The alignment coordinates of uniquely aligned reads for each sample were used to independently calculate the read depth of each annotated gene. Genes with an average of at least one uniquely mapped read across samples were tested for differential expression using QuasiSeq (http://cran.r-project.org/web/packages/QuasiSeq). The generalized linear model Quasi-likelihood spline method assuming negative binomial distribution of read counts implemented in the QuasiSeq package was used to compute a *P* value for each gene. The 0.75 quantile of reads from each sample was used as the normalization factor^65^. A multiple test-controlling approach was used to convert *P* values to *q*-values for controlling false-discovery rate^66^. For most of the comparisons, *q*-values no larger than 0.05 were considered to be differentially expressed. Owing to the strong growth phenotype of *RD26OX* transgenic lines, more stringent (*q*<0.003) condition was used to determine differentially expressed genes. Clustering was performed using the ‘aheatmap' function of the NMF package in R (https://cran.r-project.org/web/packages/NMF/index.html). Log2 reads per million mapped read values were used for clustering analysis and values were normalized for each gene by centring and scaling each row of the heatmap. The overlapped genes were identified and displayed using Venny^2.0^ programme (http://bioinfogp.cnb.csic.es/tools/venny/).

### Chromatin immunoprecipitation

ChIP was performed as previously described^23^ with modifications^67^. Briefly, 5 g of 4-week-old plants were fixed in 1% formaldehyde and used to isolate nuclei and chromatin. The chromatin was sheared with Diagenode Bioruptor Sonication System with 30 cycles of 30-s on and 30-s off in icy water bath. Twenty micrograms of affinity-purified BES1 (ref. 23), RD26 antibodies (see ‘Generation and analysis of transgenic plants' section) or IgG (Sigma, I5006) were used to immunoprecipitate chromatin, which was collected with 20 μl Dynabeads protein A (Invitrogen). Three qPCR technical repeats were used to calculate enrichment folds compared to ubiquitin control (UBQ5). The enrichment of specific transcription factors was examined by qPCR with primers from indicated regions. The averages and s.e.'s were derived from four biological repeats.

For ChIP–reChIP, chromatin was prepared from 15 g *RD26OX, BES1RNAi or rdQ* mutant plants with a modified protocol in which the crosslinking with formaldehyde was performed after tissue grinding in liquid nitrogen, and all the buffer volumes were scaled up by 15-folds compared with the published protocol^68^. The sonication and immunoprecipitation were performed as described above with BES1 or RD26 antibody. Each first immunoprecipitated chromatin sample was eluted with 75 μl 10 mM Tris-HCl (pH 8.0), 1 mM EDTA, 2% SDS and 15 mM dithiothreitol and diluted 20-folds for second immunoprecipitation with corresponding antibody (RD26 or BES1) or IgG control. The enrichment at specific regions was determined by qPCR with indicated primers as described above. The averages and s.e.'s were derived from three biological repeats.

### Other bioinformatics analysis

For promoter motif analysis, we downloaded Group 1 and 2 genes upstream 3 kb sequence from TAIR database (https://www.Arabidopsis.org/tools/bulk/sequences/index.jsp). On the basis of this sequence information, we coded in-house Perl scripts to match possible E-box and BRRE motif in upstream 3,000 bp region by searching conserved sequence ‘CANNTG' for general E-box or CATGTG for specific E-box and conserved sequence ‘CGTG(T/C)G' for the BRRE site. All the statistical analyses was done by R language (http://www.R-project.org/). We fitted a negative binominal model for fitting the frequency of E-box and BRRE domain in ‘glm.nb' function and then calculated *P* value for each comparison. The density plots were generated by R language ‘plot' function.

For re-analysis of previously published microarray data^24^,^45^,^46^,^47^,^48^,^49^, we downloaded the microarray raw CEL data from Riken and analysed the arrays using the ‘Robust Multi-array Average method'^69^ to obtain gene expression data. To analyse gene expression and compare the expression between the WT and hormone treatments, we used the linear model for microarray (limma) package from the Bioconductor project (http://www.bioconductor.org). When estimating statistical significance for log2-transformed fold-change replicates were combined analogous to the classical pooled two-sample *t*-test. To account for multiple testing, we used the Benjamini–Hochberg method, and significance level for detection is at 5%. The differential expressed genes were combined with published gene lists to obtain the BR-regulated genes by microarrays were and listed in Supplementary Data 3 and 4.

### Protein–protein interaction experiments

The Split Luciferase Complementation Assays were performed as described^50^. The coding region of RD26 and BES1 were cloned into the pCAMBIA1300-nLUC and pCAMBIA1300-cLUC constructs, respectively. Tobacco leaf transient assay was used to examine luciferase activity in the presence or absence of RD26 and/or BES1. Equal amount of Agrobacterium cells (measured by OD_600_, adjusted to same with vector-containing strain) were injected to tobacco leaves. The luciferase activities were measured from protein extracts from triplicate samples as described above. For the immunoprecipitation (IP) experiments, tobacco leaves were homogenized in protein lysis buffer (1 mM EDTA, 10% glycerol, 75 mM NaCl, 0.05% SDS, 100 mM Tris-HCl, pH 7.4, 0.1% Triton X-100 and 1 × complete cocktail protease inhibitors). After protein extraction, anti-GFP antibody (10 μl, Life Technologies-Molecular Probes, A21311) was added to total proteins. After incubation with gentle mixing for 1 h at 4 °C, 200 μl fresh 50% slurry of protein A beads (Trisacryl Immobilized Protein A-20338, Thermo Scientific) were added, and incubation was continued for 1 h. Protein A beads were pelleted by centrifugation at 2,000 r.p.m. for 1 min, and the supernatant was removed. The precipitated beads were washed at least four times with the protein extraction buffer and then eluted by 2 × SDS protein-loading buffer with boiling for 5 min. The IP products were used for western blotting with 2 μg of anti-BES1 antibody or MYC antibody (Sigma, C3956). BiFC experiments were performed as recently described^44^. BES1 and RD26 cDNAs were cloned into the N- or C terminus of EYFP vectors^70^. Sequencing-confirmed constructs were transformed into *Agrobacterium tumefaciens* strain GV3101. Agrobacteria were grown in LB medium containing 0.2 M acetosyringone and washed with infiltration medium (10 mM MgCl_2_, 10 mM MES, pH 5.7, 0.2 M acetosyringone) and resuspended to OD_600_ 0.5 with infiltration medium. Combinations of Agrobacterium were infiltrated into *Nicotiana benthamiana* leaves and examined for YFP signals 2 days after infiltration. A Leica SP5 X MP confocal microscope equipped with an HCS PL APO CS 20.0 × 0.70 oil objective was used to detect reconstituted YFP. YFP was excited with a 514-nm laser line and detected from 530 to 560 nm. The LAS AF software (Leica Microsystems) was used to obtain images with same settings.

### EMSA experiments

EMSA experiments were carried out as described previously^25^. After annealing, oligonucleotide probes were labelled with P32-γ-ATP using T4 polynucleotide kinase. About 0.2 ng probe and indicated amount of proteins purified from *Escherichia coli* were mixed in 20 μl binding buffer (25 mM HEPES-KOH (pH 8.0), 1 mM DTT, 50 mM KCl and 10% glycerol). After 40 min incubation on ice, the reactions were resolved by 5% native polyacrylamide gels with 1 × TGE buffer (6.6 g l^−1^ Tris, 28.6 g l^−1^ glycine and 0.78 g l^−1^ EDTA (pH 8.7)).

### Drought stress tolerance of BR signalling mutants

Drought stress tolerance experiments were carried out as described previously^35^ with minor modifications: different genotype plants were grown on 1/2 MS medium for 2 weeks, and then transferred to soil and grown for one more week in growth chamber (22 °C, 60% relative humidity, long day conditions) before exposure to drought stress. Drought stress was imposed by withholding water until the lethal effect of dehydration was observed on WT control or *bes1-D* plants. The numbers of plants that survived and continued to grow were counted after watering for 7 days.

### Generation of the *Arabidopsis* RD26–BES1 subnetwork

We first constructed a whole-genome network of *Arabidopsis* using the TINGe software^55^. To construct the *Arabidopsis* network, microarray data were collected from a total of 3,546 non-redundant Affymetrix ATH1 expression profiles. The data were subjected to statistical normalization and filtering, following which 15,495 genes remained for network construction. The RD26–BES1 subnetwork was then extracted from the whole-genome network using a subnetwork analysis tool, GeNA^55^. GeNA ranks each gene in the whole-genome network with respect to its relevance to a given set of seed genes and extracts a subnetwork containing the seed genes and genes of highest ranks with respect to the seed genes.

### Yeast one-hybrid assays

Matchmaker One-Hybrid System (Clontech) was used to test the binding of BES1/RD26 to *At4g18010* and *At4g00360* gene promoters following the manufacturer's instructions (http://download.bioon.com.cn/upload/month_0907/20090707_dab6285a579af1fb2ccd87zow1gx859t.attach.pdf). Briefly, promoter fragments were cloned into pLacZi (KpnI and SalI sites) and integrated into the genome of yeast strain YM4271 to generate reporter lines. Mutant reporter lines were also generated with promoter fragments in which BES1/RD26-binding sites were mutated. BES1 and RD26 were expressed in the reporter strains with pGBKT7 and pGADT7, respectively. The LacZ expression in each strain was determined by filter assays.

### Data availability

The raw RNA-seq reads are deposited to NCBI SRA with accession number PRJNA223275. All other data supporting the findings of this study are available within the manuscript and its supplementary files or are available from the corresponding author upon request.

## Additional information

**How to cite this article:** Ye, H. *et al*. RD26 mediates crosstalk between drought and brassinosteroid signalling pathways. *Nat. Commun.* **8,** 14573 doi: 10.1038/ncomms14573 (2017).

**Publisher's note:** Springer Nature remains neutral with regard to jurisdictional claims in published maps and institutional affiliations.

## Supplementary Material

Supplementary Information Supplementary Figures and Supplementary Tables

Supplementary Data 1 List of BR-induced genes revealed by RNA-seq. The BR-induced genes were identified by RNA-seq with 4-week-old adult plants treated with or without 1 μM BL.

Supplementary Data 2 List of BR-repressed genes revealed by RNA-seq. The BR-induced genes were identified in this study by RNA-seq with 4-week-old adult plants treated with or without 1 μM BL.

Supplementary Data 3 List of BR-induced genes revealed by microarrays. The BR-induced genes in seedling or adult plants derived from previous microarray studies. The original data were also reanalyzed as described in Methods section.

Supplementary Data 4 List of BR-repressed genes revealed by microarrays. The BR-repressed genes in seedling or adult plants derived from previous microarray studies . The original data were also reanalyzed as described in Methods section.

Supplementary Data 5 List of genes up-regulated in *RD26OX* transgenic plants. The genes up-regulated in *RD26OX* plants compared to WT were identified in this study by RNA-seq with 4-week-old adult plants without BL treatment (Fig. 2).

Supplementary Data 6 List of genes down-regulated in *RD26OX* transgenic plants. The genes down-regulated in *RD26OX* plants compared to WT were identified in this study by RNA-seq with 4-week-old adult plants without BL treatment (Fig. 2).

Supplementary Data 7 List of genes up-regulated in *rd26 anac019 anac055 anac102* quadruple mutants. The genes up-regulated in *rd26 anac019 anac055 anac102* quadruple mutant plants compared to WT were identified in this study by RNA-seq with 4-week-old adult plants without BL treatment (Supplementary Figure 4).

Supplementary Data 8 List of genes down-regulated in *rd26 anac019 anac055 anac102* quadruple mutants. The genes down-regulated in *rd26 anac019 anac055 anac102* quadruple mutant plants compared to WT were identified in this study by RNA-seq with 4-week-old adult plants without BL treatment (Supplementary Figure 4).

Supplementary Data 9 List of genes up-regulated by drought stress. Drought induced genes (combination of 2-day and 3-day dehydration treatment data) were from previous study by microarray analysis.

Supplementary Data 10 List of genes down-regulated by drought stress. Drought induced genes (combination of 2-day and 3-day dehydration treatment data) were from previous study by microarray analysis.
