# Supplementary material for: RD26 mediates crosstalk between drought and brassinosteroid signalling pathways
Source: Nat Commun. 2017 Feb 24;8:14573. doi: 10.1038/ncomms14573 (PMC5333127; doi:10.1038/ncomms14573)
Supplement: Supplementary Information — Supplementary Figures and Supplementary Tables [file ncomms14573-s1.pdf]

Supplementary Information

Supplementary Figures and Tables

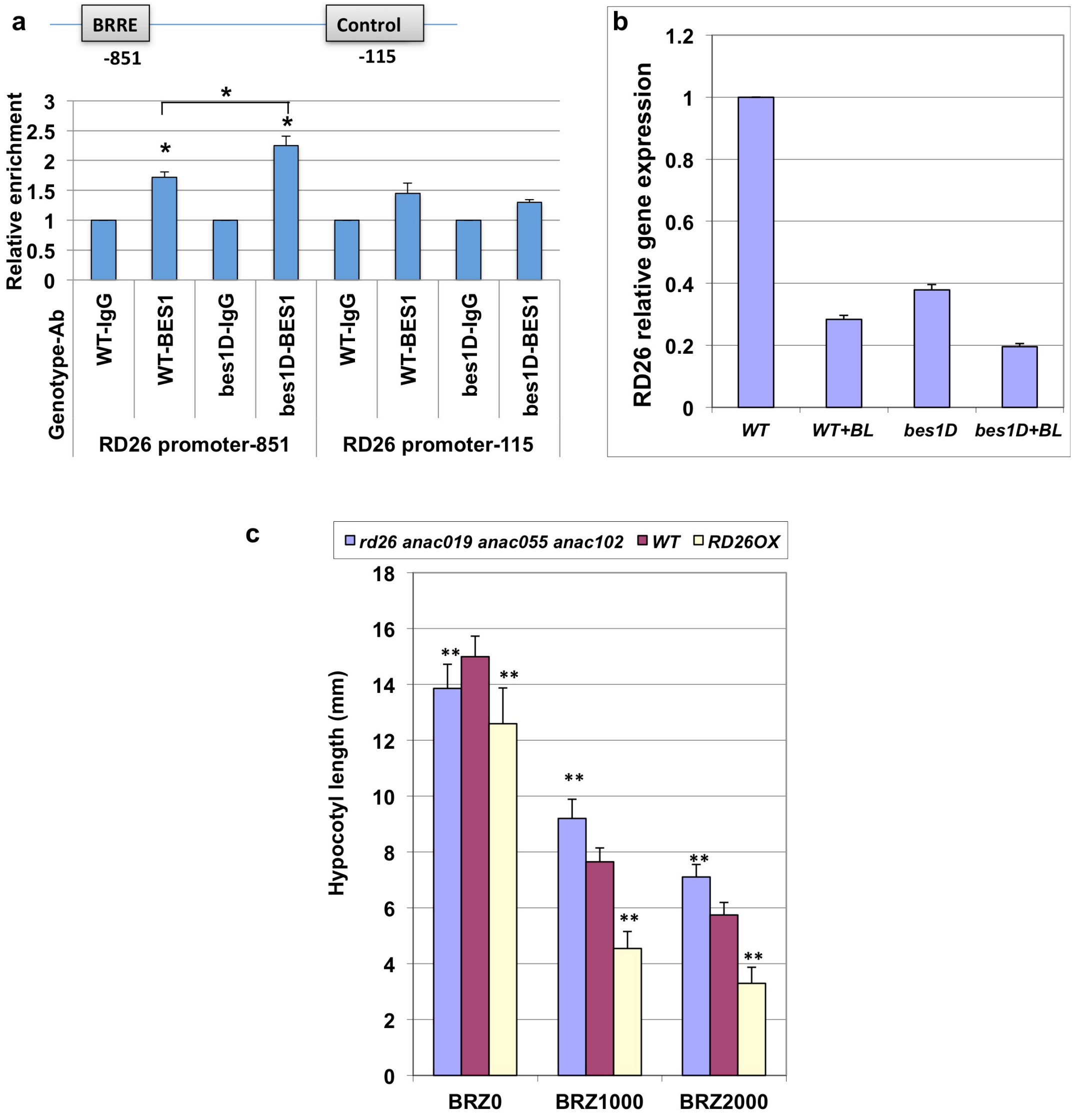

Fig. S1: *RD26* is a target of BES1 and functions as a negative regulator in the BR pathway. (a) BES1 targets the BRRE site on RD26 promoter. ChIP was performed with anti-BES1 antibodies in WT and *bes1-D* adult plants. The bindings of BES1 at BRRE site (-851) and control site (-115) of the *RD26* gene promoter were examined by qPCR.
(b) The expression of *RD26* was examined by quantitative qPCR in WT and *bes1-D* plants with or without 1,000 nM BL treatment for 2.5 hr.
(c) BRZ responses of RD26 overexpression (*RD26OX*) and *rd26 anac019 anac055 anac102* quadruple mutants. The hypocotyl lengths of 5-day-old dark-grown seedlings in the absence or presence of different concentrations of BRZ. Error bars indicate s.d. (n=15-20). The difference was significant based on Student's t-Test (*p<0.05; **p<0.01).

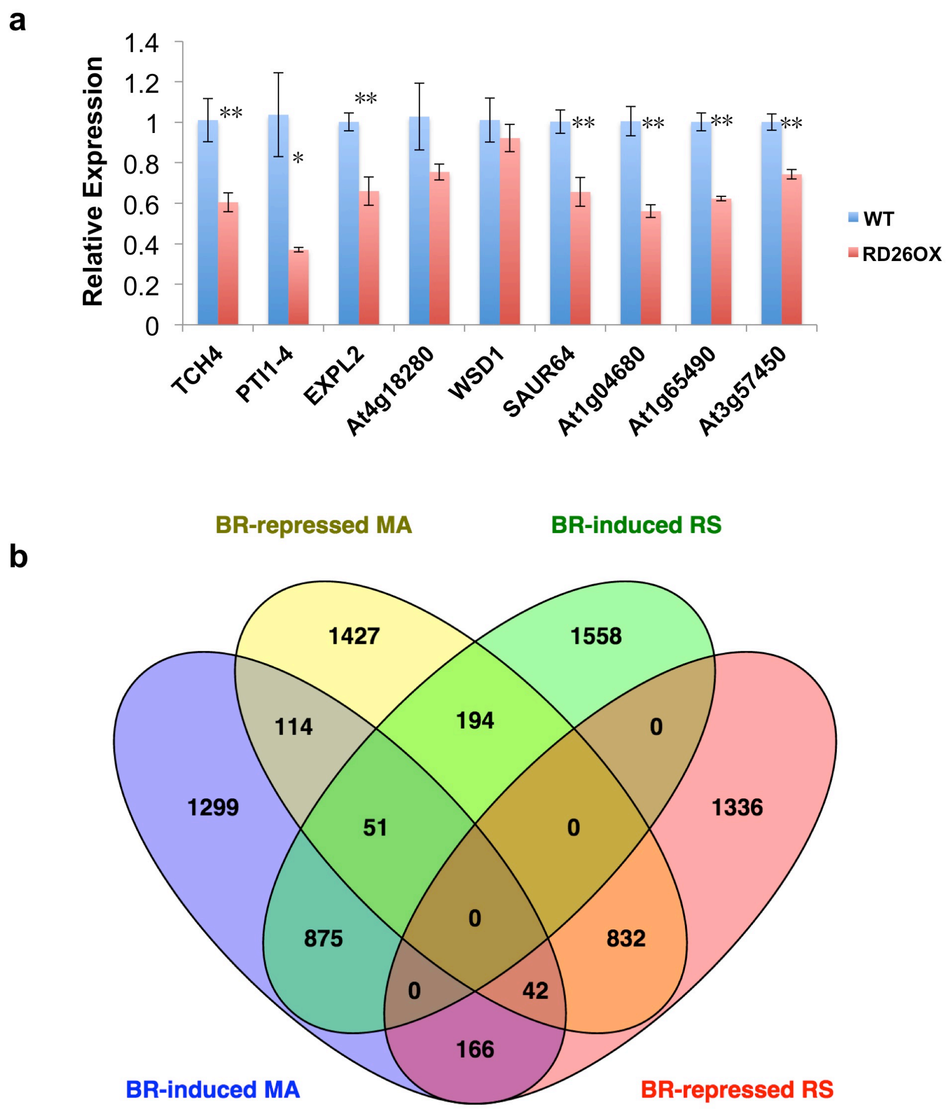

Fig. S2: (a) The expression of BR-response genes in *RD26OX Arabidopsis* plants.
The expression of the selected genes was examined by qPCR using RNA prepared from corresponding 5 week-old plants. The significant difference test was based on Student's t-test (*p<0.05, **p<0.01, n=3).
(b) Overlap between BR-regulated genes identified by RNA-seq (RS, this study) and previously published by Microarrays (MA). Venn diagram shows that about 43% of genes overlap between those identified by RNA-seq with adult plants and those identified by microarrays from seedlings or adult plants [24, 45-49]. Note that a small portion of genes can be either induced or repressed, likely depending on physiological conditions of BR treatments.

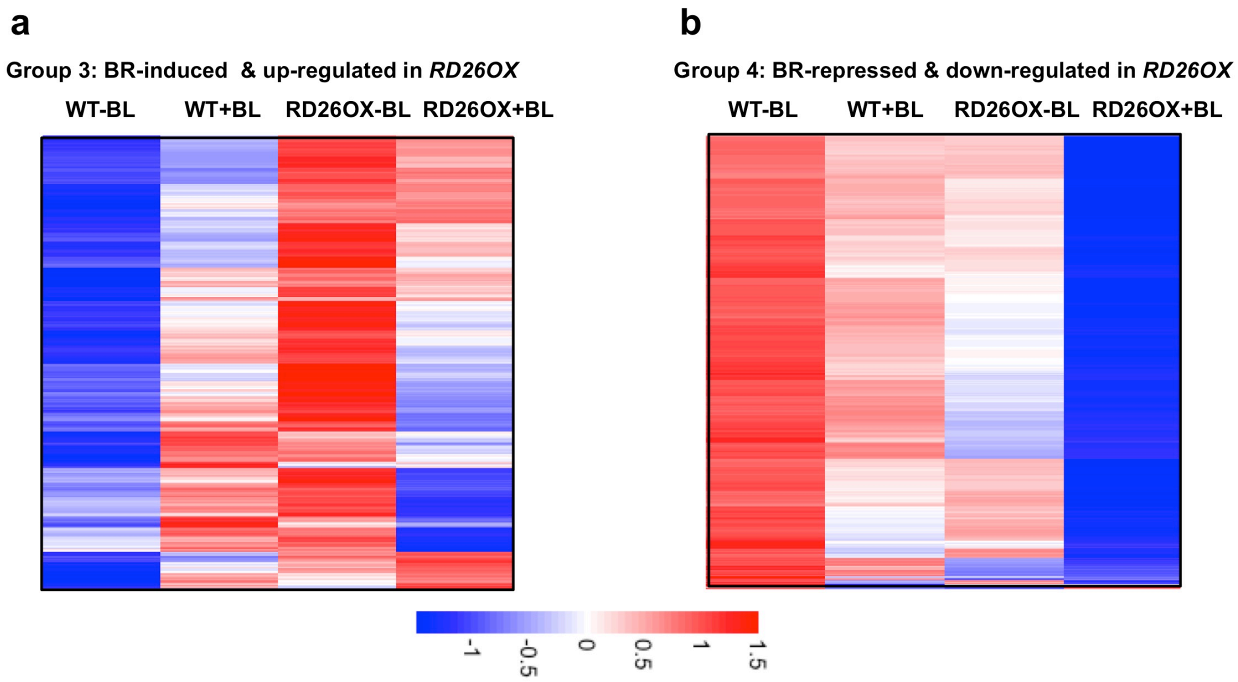

Fig. S3: **Clustering analysis of Group 3 and Group 4 genes.**
(a) Group 3 genes expression in WT with or without BL treatment (lane 1 and lane 2) and in *RD26OX* transgenic plant with or without BL treatment (lane 3 and lane 4).
(b) Group 4 genes expression in WT with or without BL treatment (lane1 and lane 2) and in *RD26OX* transgenic plant with or without BL treatment (lane 3 and lane 4).

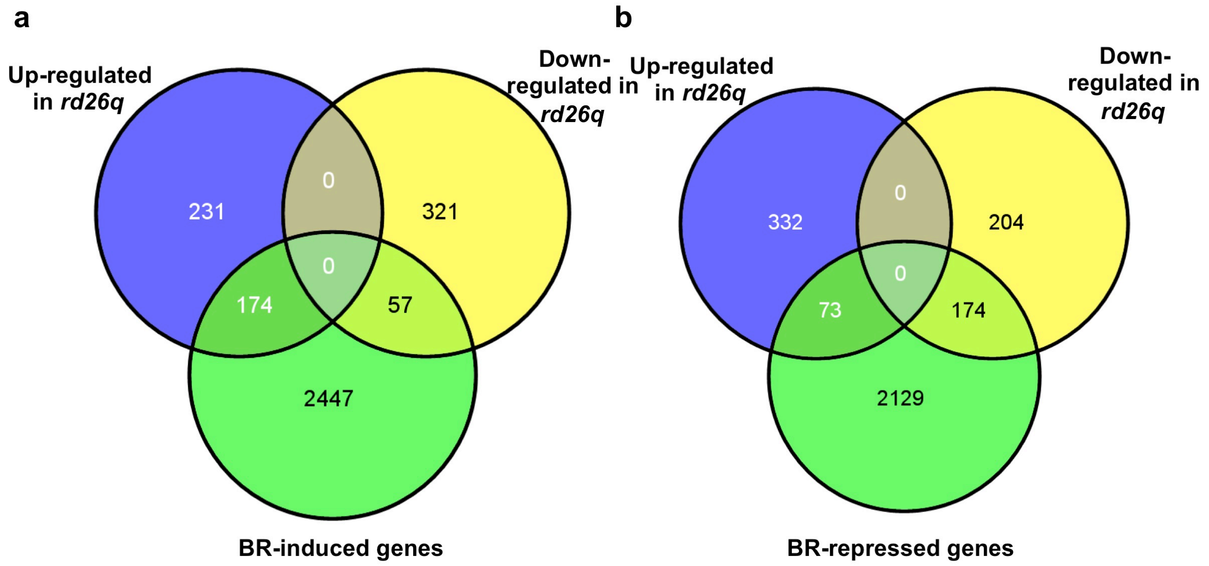

Fig. S4: Overlap between BR-regulated genes and genes affected in *rd26 anac019 anac055 anac102* quadruple mutant.

Venn diagrams show the overlap genes between BR-induced (a) or BR-repressed (b) genes affected in *rd26 anac019 anac055 anac102* quadruple (*rd26q*) mutant-regulated genes.

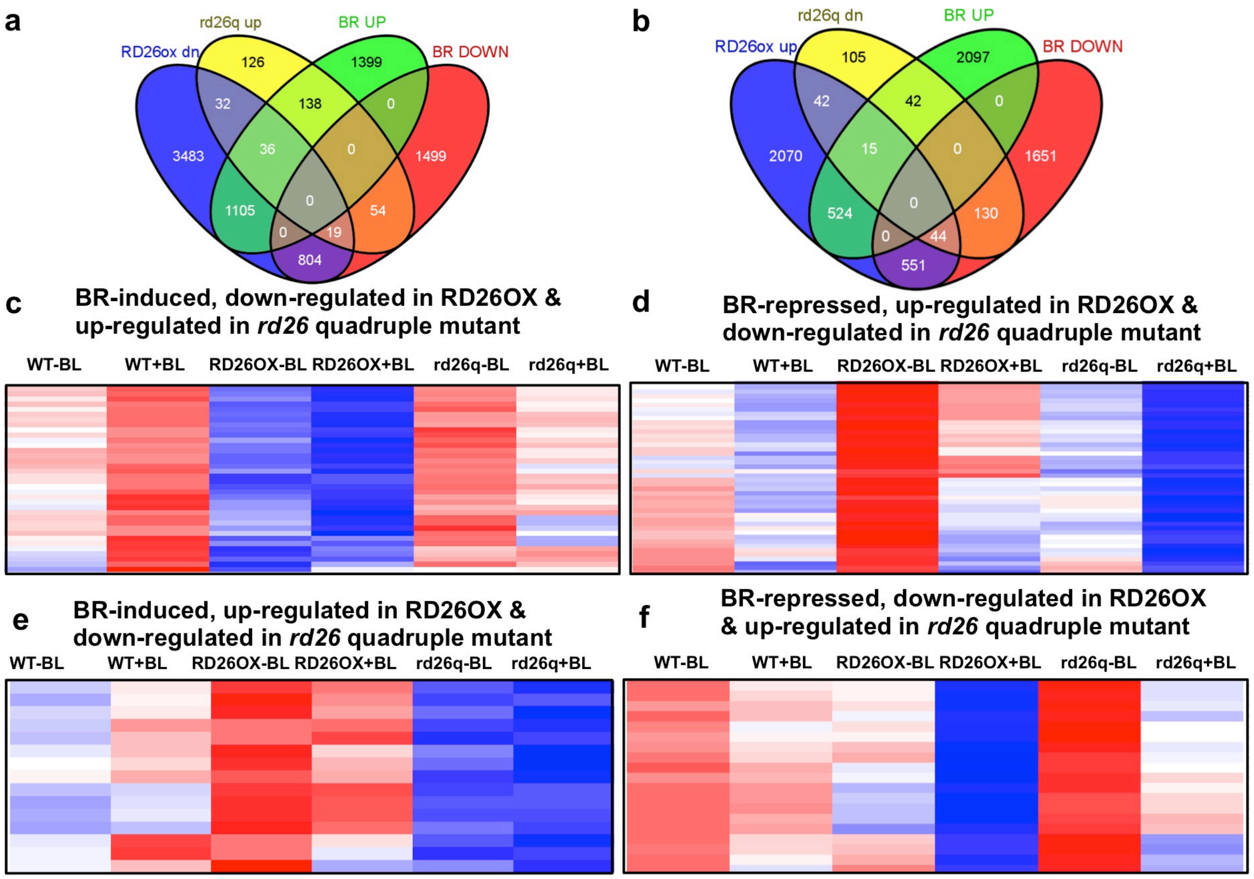

Fig. S5: Overlap between BR-regulated genes and genes differentially affected in RD26OX and in *rd26 anac019 anac055 anac102* quadruple mutant.
Venn diagrams show the overlap genes between genes up-regulated in *rd26q* and down-regulated in *RD26OX* with BR-regulated genes (a), genes down-regulated in *rd26q* and up-regulated in *RD26OX* with BR-regulated genes (b).
(c-f) Clustering analysis of BR-regulated genes affected in opposite ways in *RD26OX* and *rd26q* quadruple mutant.

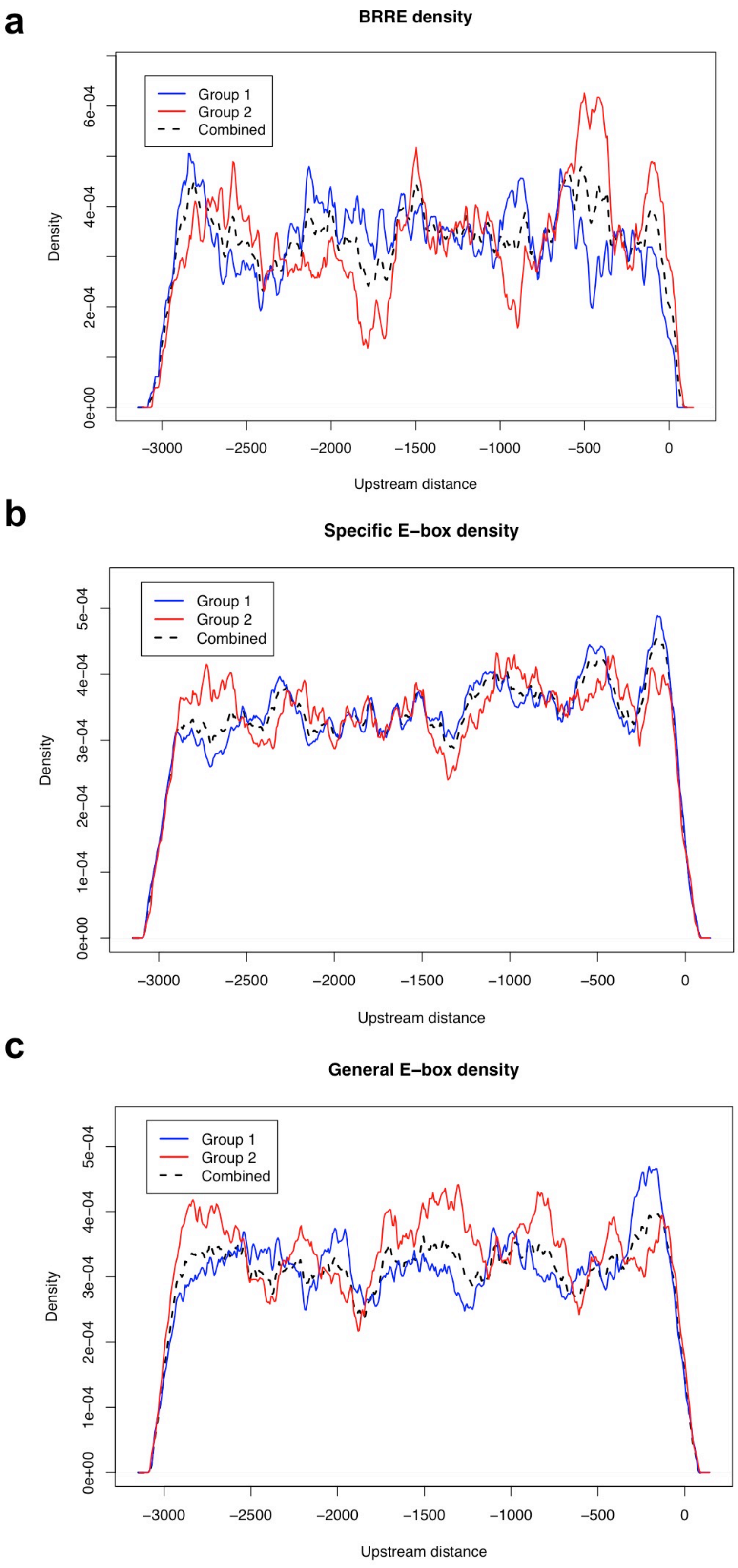

Fig. S6: Different BES1 binding sites are differentially enriched in Group 1 and Group 2 BR-regulated genes.

(a) BRRE sites (CGTGT/CG) are enriched in Group 2 genes, especially in the -500 bp promoter regions relative to the transcriptional start sites.
(b) A specific E-box, CATGTG, which is often found in BR-induced genes, are enriched in Group 1 BR-regulated genes in the -500 bp promoter regions.
(c) The general E-box (CANNTG) are also enriched in Group 1 BR-regulated genes within -500 bp promoter regions. Although the general E-boxes are also enriched in Group 2 genes between 1,000-3,000 bp of their promoter regions, the functions of these E-boxes are not clear as BES1 and BZR1 mostly bind to -500 bp promoter regions to regulate gene expression [23, 24].

a At4g18010 Promoter Sequence

ttgtgaatcaaactaatttatttaagtagctttggtcaaccgaaactttcctaatgaccttcgttacgtcctctatccgc
aaattgcattgcatgggcgtcagtttttttttcctttttcttaataacgcgtcaatttattttatgatgatgattatgtttagttga
P1 aataatcttcagttattaattagatatttcttggtaaatatttttacaaatatgctgtcagaaattcaaagttatgtatttcca
taccatattggactccatactttatatcttctcagttgagtatccttcaaattcacctgttttggaaattggaaatataattta
gttttagaaatgttttaagttaaaacataacagttaa**caaagaaaagatatagacaattttaaga****cttttaaatgtg**
agggattctcactttttcgttgttttctaatggttccaattcatagttatcgtataatattatttaacgaaacattagtcaaa
P2 taaataatctatctattcgccgactgtatggctgaaaatattagctggagctagactcgtattgaaaaaattaatccat
atatttgttgcctaggagagtgacattttgaccttttaataacataccaaaaaggccgtttatttaatatattatccagat
agcagcgttgaatcggattttggttggttttggtttatgtgttttagatttttggttaaagcaaaccactaacttaagtcagg
tcaaagtatttcagcaaa**actaattctcaaataatcacttttactccg****caaaactgcttgtcaaaatatagtt**ttg
acttccaacgagccatttttgctaattcagtttaactagtatacgcaaaaaaaaaaaaatgtttagataaattaaaattt
P3 cggttaacttttataaaaaagtttcagtctgtctgtttgattcggtttactttaactaaatgccactatcttaaaagatgaa
gtaaacatttataataattcggtatggtcggtacaatcagtctagtaaaaccgaattgagaaaaccaggtcggttcg
aatatatccaa**aaaccgaaaacacgtgtgagaaagaagaaa**tcagggaattaaatctgacagtctttatagttt
tctttaatatgtcttccccaaaaaatcaaattatgaataaaaaa**agtggatcgcaagttgcag**tttggttctttacgg
gcaacgaaggctgttaaataacagccatagcttttcttcgaaacgaacccaaaaaataatatcagagaattttcat
cattacacctttttttttcttttgcctttgcctttgccttcacacgtcttcttgtttgtactctccactttttttttctttgtgtcctttcttct
tgttcttgctctagttcgtgtctcttctttaactctctgtcactattataaatatcaaactctgacattttcttctgttcaaattctc
ctcgaaactcccccaaa

b At4g00360 Promoter Sequence

tctcttgatacaatgcatatagaaactgacaaataatcgaagaaattgtacttgagcccattacgactattgaa
aattctgattttggatgaattcagcggaaactacaaatttaagagtactttatgtgtataatatgaagcctatatatat
P1 agaattactaatgtaataaaataagaaccggtgaaaagggggacaagatcacaaggttttcgcattcagtgcct
ttacagagttatatatttgatgatgttattgcttacttgcctaatagtactatactactatatatatctaaggtaacacatg
tatatatatgtcacatagacattactagtatat1028**attatgtacttctatcatatatttatgatattgcagttgca**
gcgtacacaagtcagctccttttgacttttacatctcatgaatgcattgccatgacatctaatcttactcgagatttgt
gcatgcacattattcacttttgtcttttgcaatttttgttattgtaaaaaaaggaaaaacaaatgtaaaagagagaga
P2 gaccagaaaggtctaactaaacctaaagagtcaatgaaatgtgtttctttcttgtgggattaatcaattcactcttta
acacttctttataccattgaagaaattagatgaaagagtcacgagttgcttacc**aaatccctcacaagaattga**
gaactgataaaccaaattgagaagattaaatatcacgtctccttttgatctctattataattaatcgaaaataaaa
ataagagttttcaacaaaacgtgatcattggtttacgatcacttgcaaagtcaaacctaaaacgtagcattagtac
P3 actaaccttaaatactaattatatcatgcaaaccctaatgtc**attacctaactatacatgtgtaatgtgttc**aaca
gatcttcttaacccacattagatcaatattaaacaataaaaagattcttatatattctactacttacttcttcttattccca
tccatatttttctgtgcctttta**ggttctcaactaatctcatttaatttagct**agcacacagagaaacacacacgtat
ataaataatatgataacacacaaaaagactcatatatataaataattagagtcattaaatgtggattcatcattaa
atgaaacaactcttcttctctgtacaatttctcttcacaccttcaccaaattctttgacttcaaaaatcttataaaatttat
atatctccaaaaccataaaaccaaaacgagttttcacaaataaattacttagttgaaatttcaaatctcattcaatta
gggtacactctctcaacaatccacattaatgag**ggttgctgcttctgatggcta**

Fig. S7: Promoter sequences of At4g18010 (a) and At4g00360 (b) used in the study.
The DNA sequences from 5'-3' are shown. Three promoter fragments (P1, P2 and P3) used in yeast one-hybrid assays are indicated and color-coded. The primers used to clone each promoter fragments are also indicated. BRRE site (CGTGTG) in At4g18010-P3 (a) and CATGTG E-box in At4g00360-P3 (b) are indicated in red.

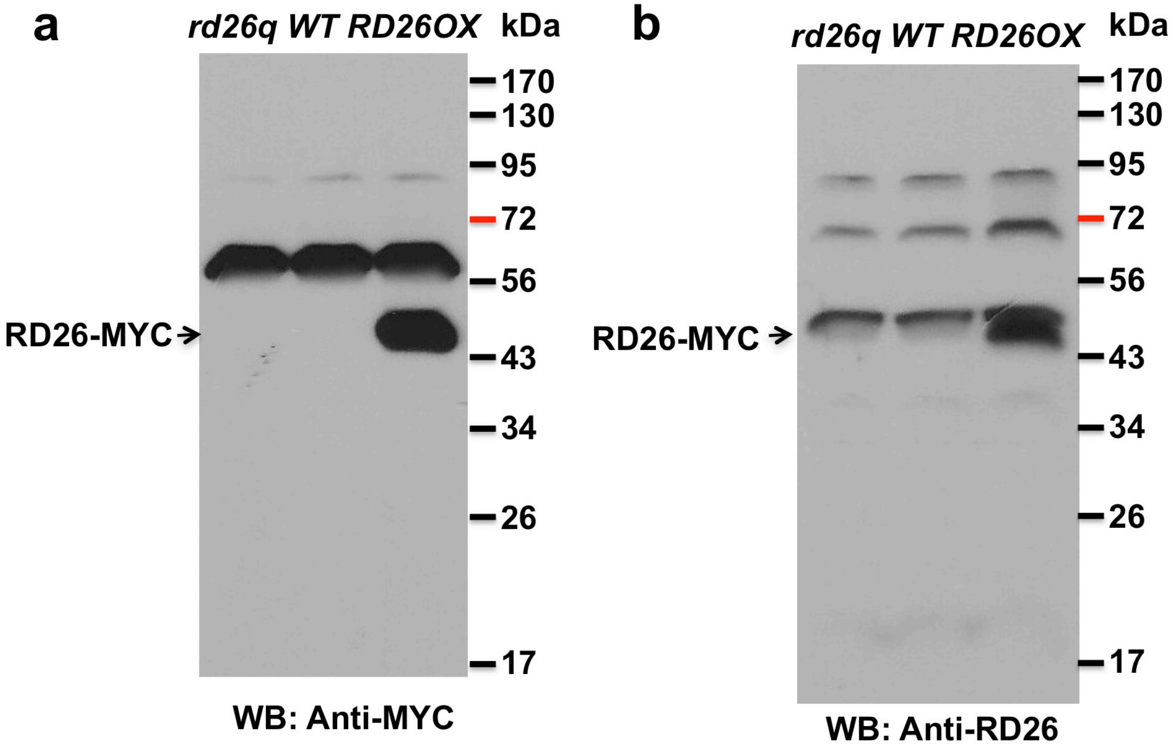

Fig. S8: Validation of anti-RD26 antibody.

(a) A Western blot of protein prepared from *rd26q* quadruple mutant, WT and *RD26OX* plants with anti-cMyc antibody (Sigma, CS3956).

(b) A Western blot of protein prepared from *rd26q* quadruple mutant, WT and RD26OX plants with anti-RD26 antibody. The RD26 antibody detects overexpressed RD26-MYC in the transgenic lines, but not in *rd26q* quadruple mutant or in WT plants likely due to the fact that *RD26* expression is induced by drought stress.

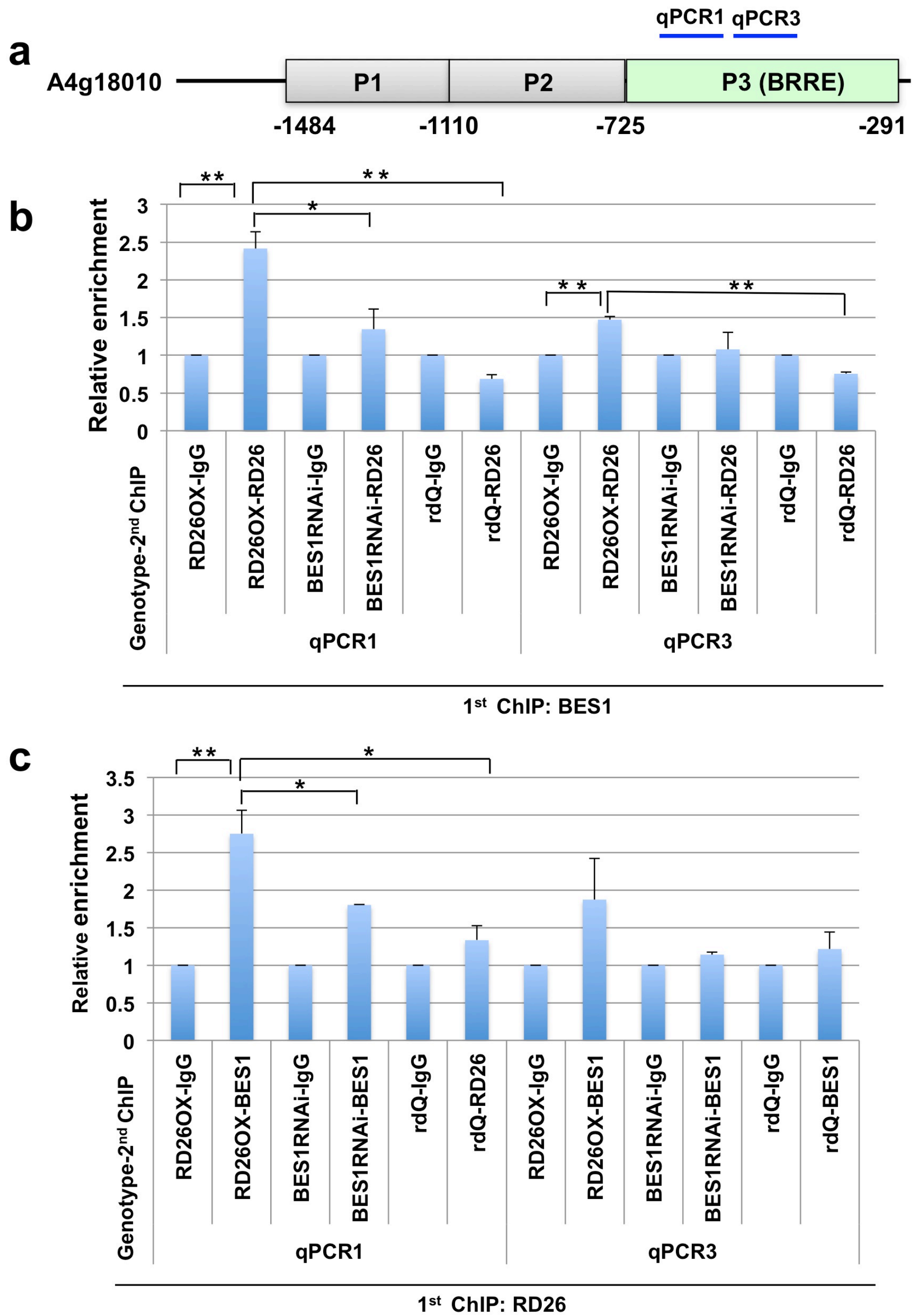

Fig. S9: BES1 and RD26 bind to target gene promoter as revealed by ChIP-reChIP.
(a) At4g18010 promoter structure shows two qPCR primer-pairs (qPCR1 and qPCR3) used. (b-c) RD26 and BES1 can bind to At4g18010 simultaneously as revealed by ChIP-reChIP. Chromatin prepared from *RD26OX*, *rdQ* and *BES1*RNAi plants was firstly immunoprecipitated with BES1(b) or RD26 (c) antibody. The first ChIP products were immunoprecipitated for 2nd ChIP with either RD26 (b) or BES1 (c) antibody as well as IgG and detected using qPCR primers. The qPCR1 primers appear to work more effectively than qPCR3 primers. Error bars indicate s.e.m. (n=3).

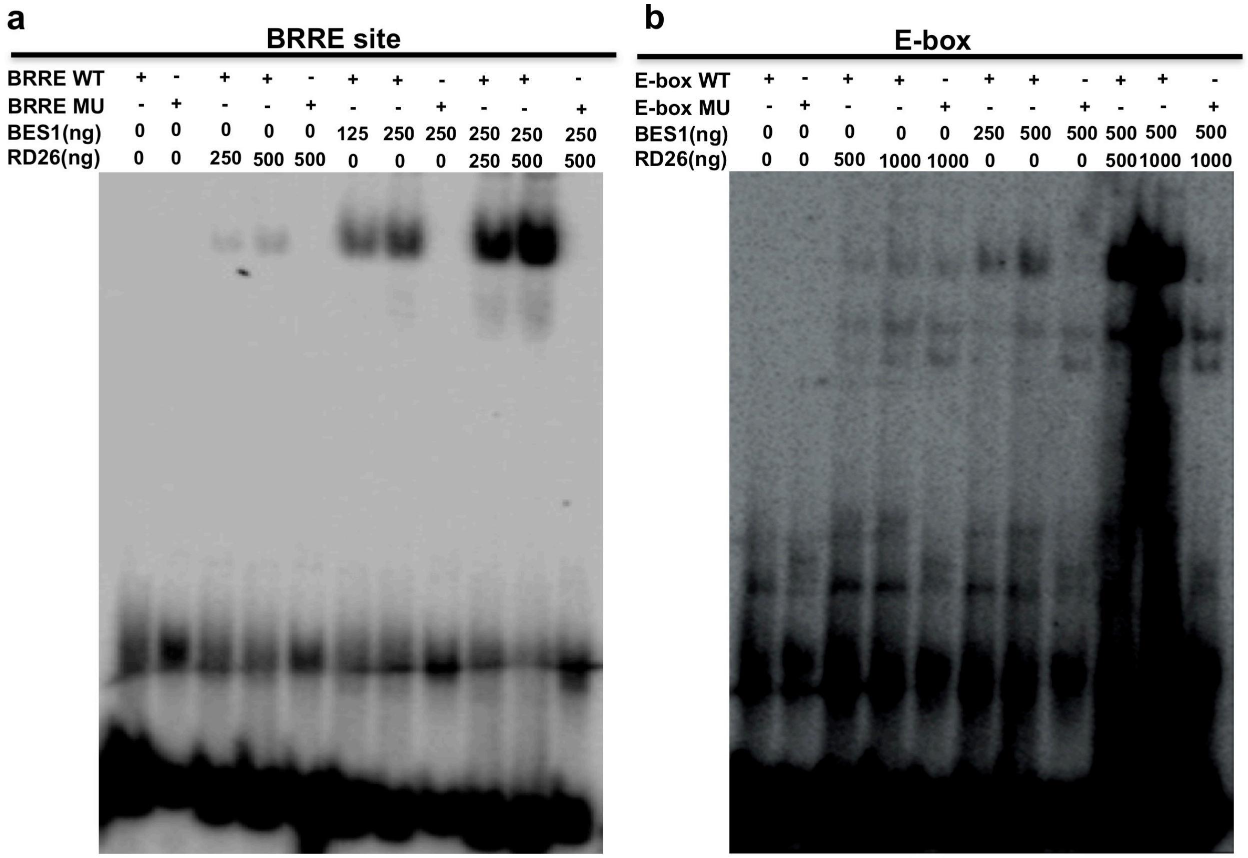

Fig. S10: BES1 and RD26 individually bind to E-box or BRRE site, but they together displayed strong synergistic binding on both sites. The full images for Fig.5b and Fig. 5c are shown.

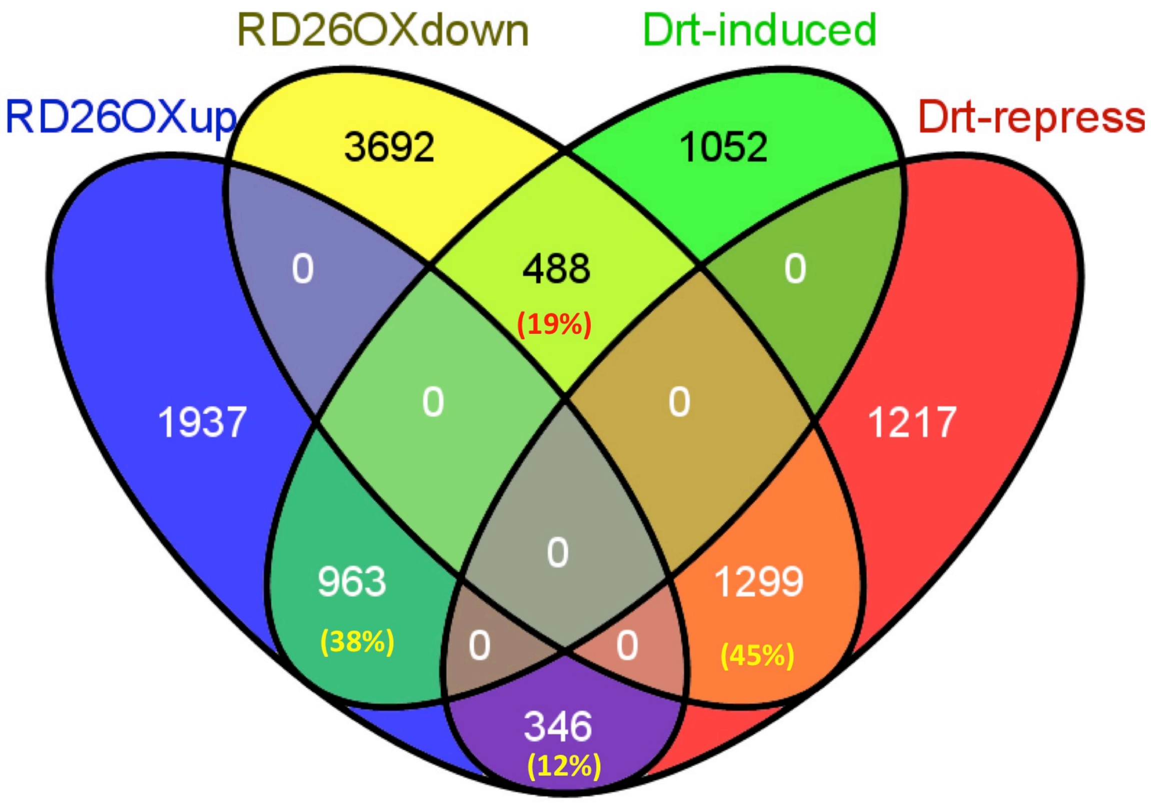

Fig. S11: RD26 mediates the expression of a large portion of drought-responsive genes. Venn diagram shows the overlap genes between genes either up- or down-regulated in RD26OX as well as drought (Drt)-induced or drought-repressed genes. The drought-regulated genes are derived from [51].

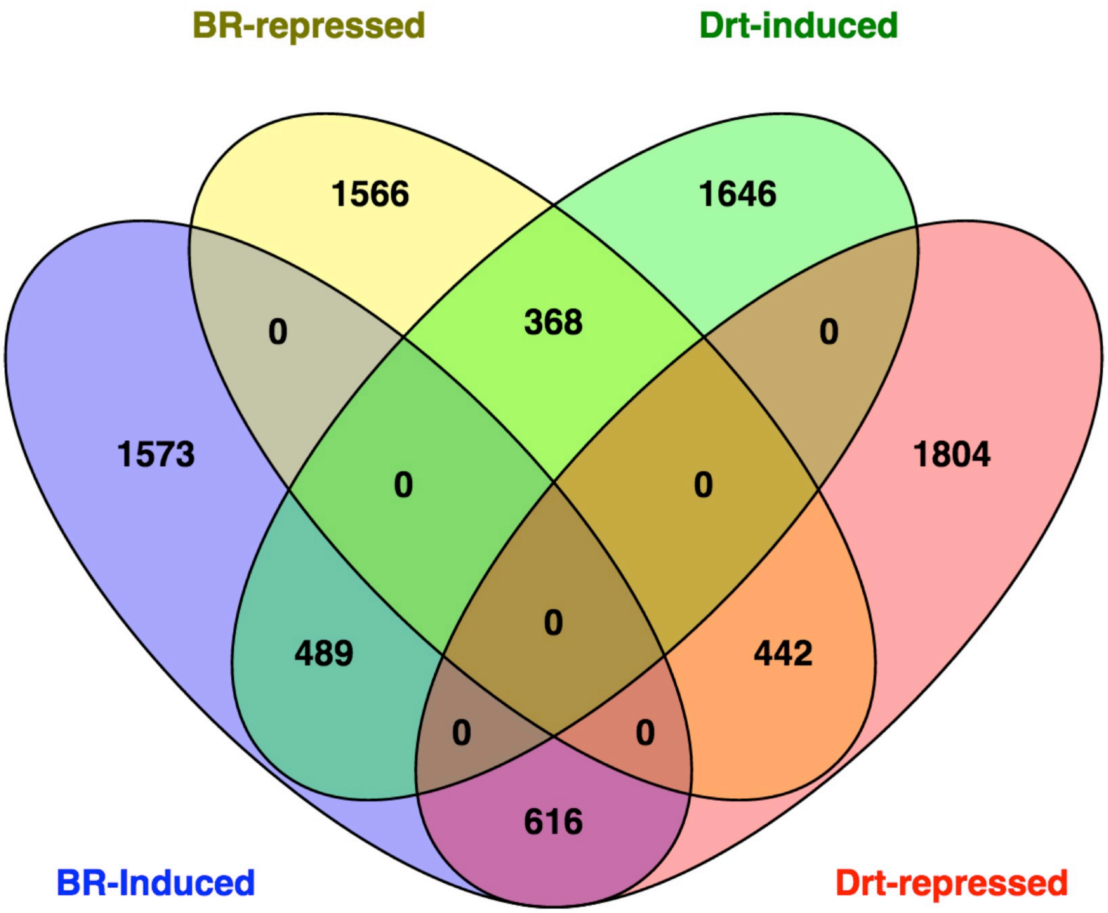

Fig. S12: Overlaps between BR-regulated and drought-responsive genes.
Venn diagram shows the overlap genes between genes either BR-induced or BR-repressed as well as drought (Drt)-induced or drought-repressed genes. The drought-regulated genes are derived from [51].

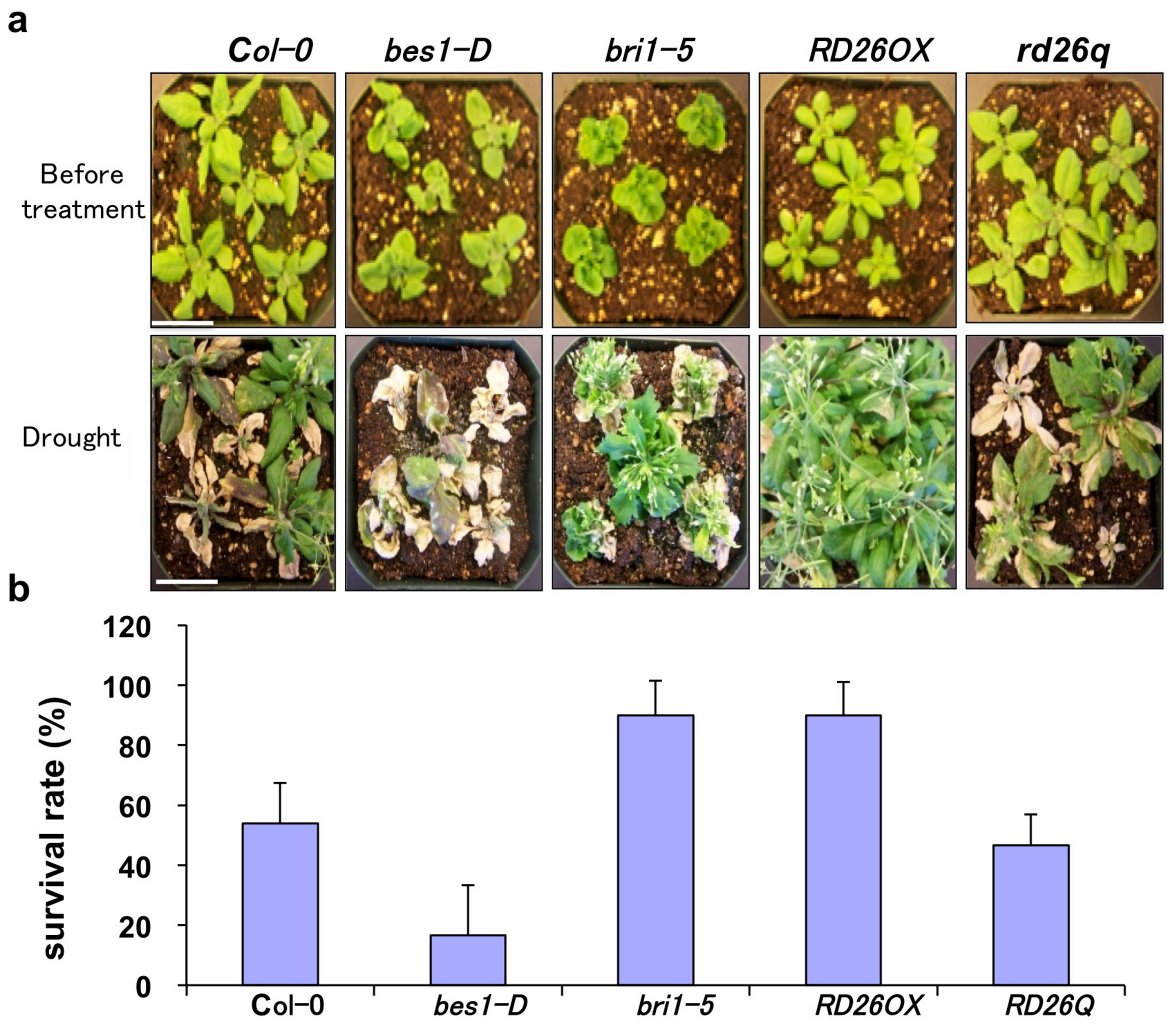

Fig. S13: The drought response phenotypes of BR and *RD26* mutants.

(a) Drought assay (4 week-old plants were withheld water for 2 weeks, and then re-watered for 3 days.) for *Col-0, bes1-D, bri1-5, RD26OX* or *rd26 anac019 anac055 anac102* quadruple mutant *(rd26q)* plants. For each line, 20 plants were tested. Selected pots with plants before drought treatments and after drought treatments are shown. The survival rate for two biological repeats are shown. The bars represent 2.5 cm. (b). The experiments were repeated three times with same trend.

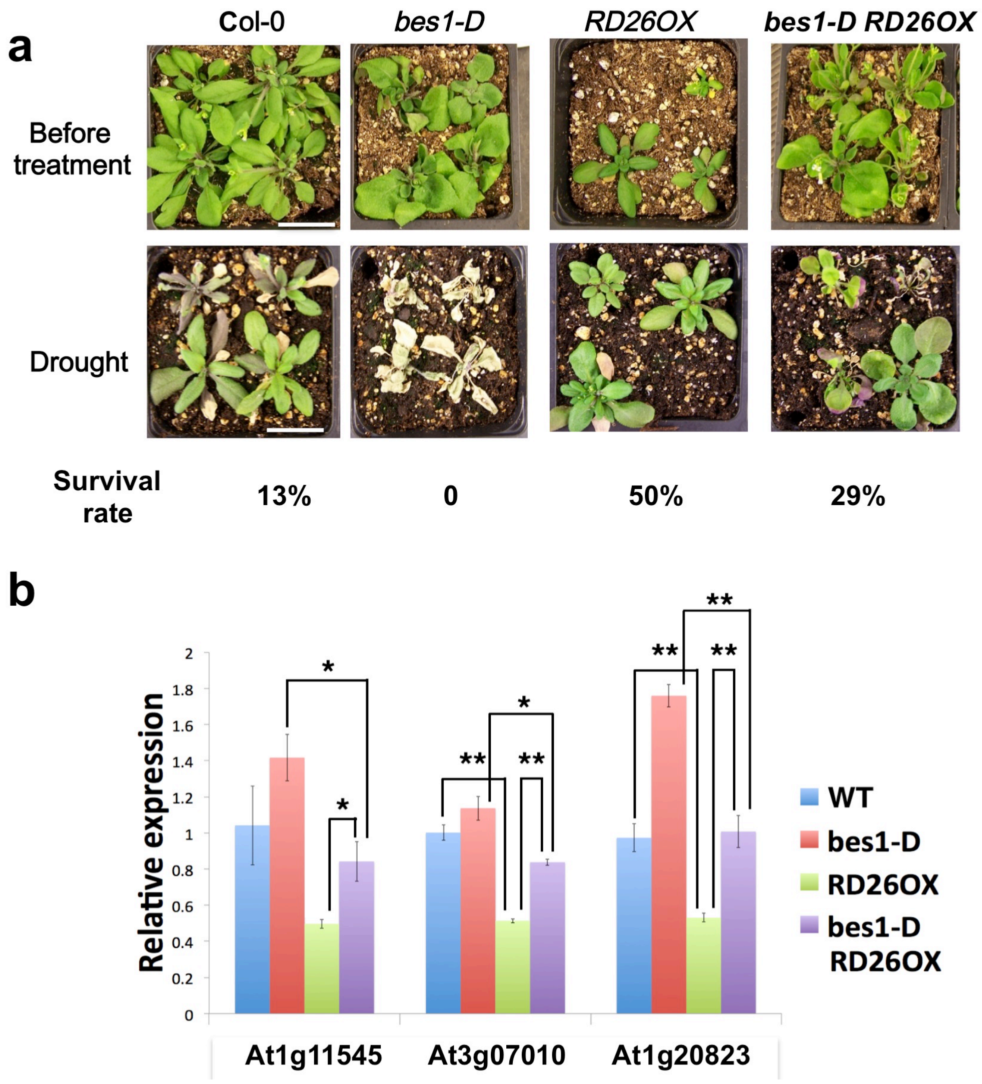

Fig. S14: *RD26* overexpression suppresses *bes1-D* phenotype in drought response.
(a) Drought assay (4 week-old plants were withheld water for 2 weeks, and then re-watered for 3 days.) for *Col-0, bes1-D, RD26OX, or bes1-D RD26OX plants.* For each line, 32-40 plants were tested. Selected pots with plants before drought treatments and after drought treatments are shown. The bars represent 2 cm. The experiments were repeated three times with same trend.
(b) The expression of several BES1 and RD26 regulated genes in *Col-0, bes1-D, RD26OX, or bes1-D RD26OX plants.* The expression of those genes were examined by qPCR using RNA prepared from corresponding 5 week-old plants. Error bars indicate s.d. (n=3).

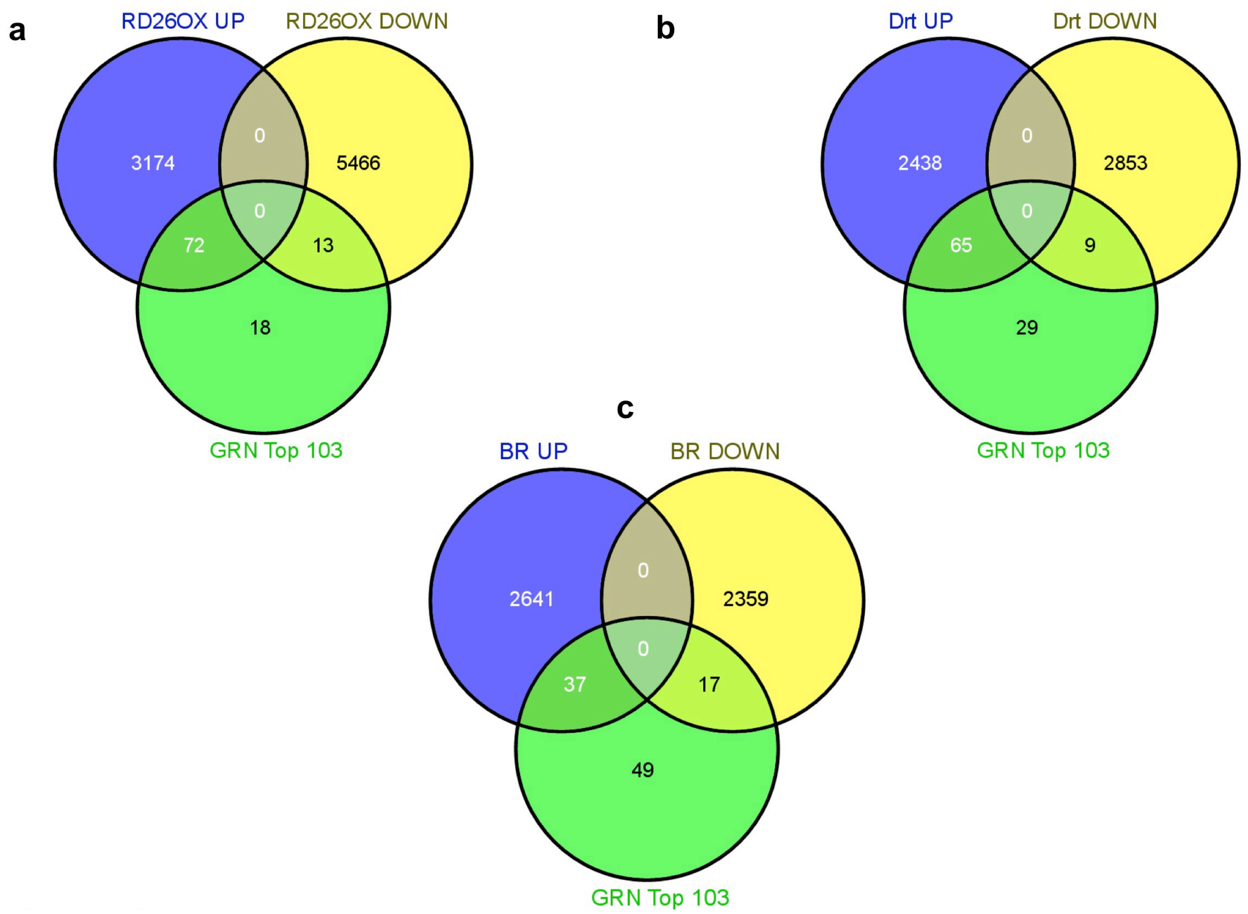

Fig. S15. Validation of GRN by differential gene expression in RD26OX, and by drought or BR- regulated genes.

Venn diagram shows the overlap genes between genes in the top 103 of the derived GRN (GRN top 103) and genes either up- or down-regulated in RD26OX (a), genes regulated by drought (b), or genes regulated by BRs (c).

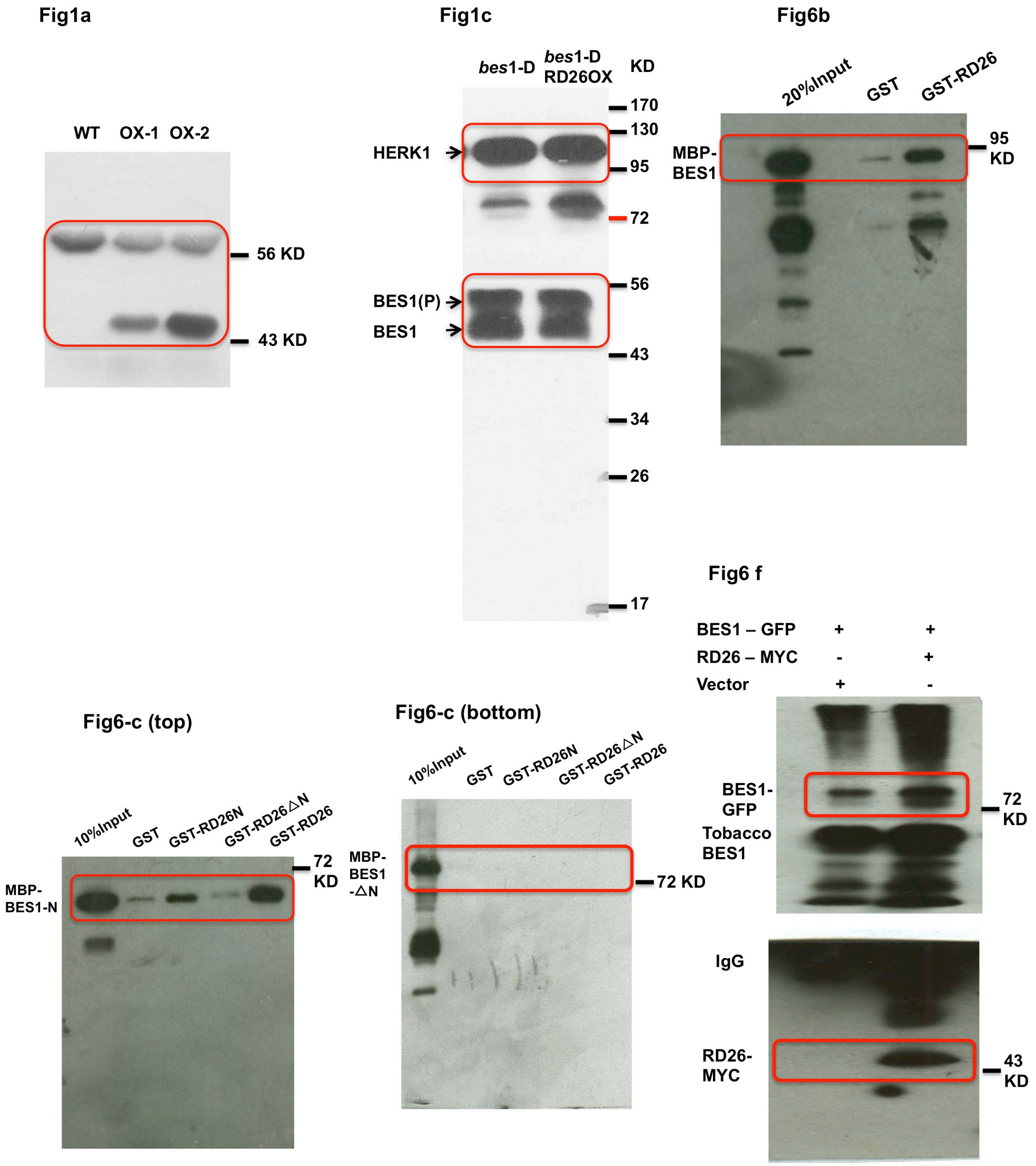

Fig. S16. Uncropped images of Western blots present in this study.

Table S1. Frequencies of specific promoter elements presented in Group1 and Group2 gene promoters

	Specific E-box (CATGTG)	General E-box (CAXXTG)	BRRE (CGTGT/CG)
Group 1	0.17*	1.73	0.05
Group 2	0.13	1.67	0.12*
Control	0.12	1.65	0.07

The sequences in 500 bp upstream of the selected promoters were used to search for indicated promoter elements. The frequency of indicated promoter element in each one promoter is indicated. The control used 1000 randomly selected genes that are not in Group 1 or Group 2. The significance of enrichment compared to control was calculated by fitting negative binomial model and indicated (* $p<0.05$).

Table S2. The DNA primers used in this study

RD26LP	AGTGATCGAGTGCTTCAGGAC	GENOTYPING
RD26RP	ACTCGTGCATAATCCAGTTGG	
ANAC019LP	TCAATGAACTCAAGGGATTGC	
ANAC019RP	ATGCGGTTTGGGTTAGAAAAC	
ANAC055LP	TAAACGATGAGCGATAGCGAG	
ANAC055RP	AAAGGAACCAAAACCAATTGG	
ANAC102LP	TAATCGTATGACCCGACTTGG	
ANAC102RP	TCTATCTTTGCCGGAGATGTG	
RD26BamHI	CGCGGATCCATGGGTGTTAGAGAGAAAGATCCGTTAG	PROTEIN EXPRESSION
RD26SacI	GCCGAGCTCTCATTGCCTAAACTCGAATGTTTGACCCG	
RD26SalI	GCCGTCGACTCATTGCCTAAACTCGAATGTTTGACCCG	
RD26NSalI	CGGGTCGACTATTAAGCGATACTCGTGCATAATCC	
RD26‣NBamHI	GCGGGATCCGAACATTCTCGTAGCCATGGAAGC	
gRD26NAsp718	CGCGGTACCATCTCTCTGTGAACAAGAATTCTCCACGTTCAC	TRANSGENIC PLANT
gRD26CSal1	CGCGTCGACTTGCCTAAACTCGAATGTTTGACCCGAAACACC	
RD26CHIP1F	TCCCAACACGTGTACAATTCA	BES1 ChIP
RD26CHIP1R	AAAACAAATGGCACTAAGACGTT	
RD26CHIPCF	TTGTCCAAAAGATCGACGAA	
RD26CHIPCR	CTTCGATTCCTCAGCAACCA	
RD26RTF	GGCACTAAAACCAACTGGATTATGCACGAG	GENE EXPRESSION
RD26RTR	GGAGTAACAGCTTGTCTCTGAGATCCAG	
ANACO19RTF	GCTCCTAAAGGTACTAAAACCAATTGGATC	
ANAC019RTR	CCATTATCGTAAACTTGTTTTTGTGCAC	
ANAC055RTF	TTGGATTATGCATGAGTACCGTCTCATCG	
ANAC055RTR	CCATTGTTGCTGTATTCACGACCACTCG	
ANAC102RTF	CGAGTATCGTCTCGCTAATGTCGATCGATC	
ANAC102RTR	ACGTACTCATCTTTTCCGTCGGTTTCTCAG	
BOS1RTF	TTCATGAATTACGACTACAACAACAA	
BOS1RTR	AGAACCAGAATTCTTCATCAGTTTCT	
ERD1RTF	ATTGATCATAATGACCTCTAATGTCG	
ERD1RTR	ATCTTCAACAATCTCTGTGACAGTTC	
AT1G29395RTF	CAGAAACCATTCCTCTCTCTTAAACT	
AT1G29395RTR	ATACACCATACTCTCCCTTAATCCAG	
AT3G62650RTF	GGAGAGGATACGAGAAGCTTGAT	
AT3G62650RTR	CACCATCAGTATCGACTTGTAAATCT	
AT1G10070RTF	GTCTATGCATCTCCAGTTGGTAACTA	
AT1G10070RTR	GCCTTCTCTACTACCTGATAACCTTG	
AT1G20823F	TGTTGCCAGGTGTCACAAGT	
AT1G35230F	CTCCCTCAGCTCCTACCACT	
AT5G52760F	CATGACCGCAAAGAACGCTG	
AT1G20823R	CTTCGCCTTGCTTGATTCGG	
AT1G35230R	GGAGAGCCACTTAGGGGAGA	
AT5G52760R	TCGAGAAACGGTGACAAGCG	
AT4G01870F	CATGTGAGTTTCAATAAAGATGGTG	

AT4G01870R	CGTCTAATTTCACAACGTACAAATC	
AT1g11545F	AATCGGGAGATGCGACATTC	
AT1g11545R	CGTGTAGCCCAATCGTCAGC	
AT3G07010F	CTAAGGAGGTGACTAAGAGAGAGTAC	
AT3G07010R	CGACGAGTGAGGATGATTTG	
AT1G22400F	GCGGATCAACGCTGGAGATA	
AT1G22400R	AACTCTCATCTTTTAGCGGACA	
AT5G17860F	CCGCCTGGTTGTTTGTTCTG	
AT5G17860R	TGCCAAGAGAGAGAAGCGTG	
AT1G65490F	ACGCAACGAAGAACGAAATGG	
AT1G65490R	GGATTTCCCCAAAACGCAAGT	
AT1G04680F	TAAAGAGGTGACGAAGAGAGTGG	
AT1G04680R	GTGGTGGGGTAGTAACTATGAGG	
AT3G57450F	GAAATACACGGAGATGTTGGAC	
AT3G57450R	TACACTTGAGAAGATTTGATACCG	
TCH4F	GGTTCCCTCAAGGTCTTCCTA	
TCH4R	AAAAGCACATTGTAACAAAGAGAATA	
PTI1-4F	AGTCACATGGGCTACACCTAAAC	
PTI1-4R	CTTCCCCTGGAGCTACGGC	
EXPL2F	TGTCGATATTGAATACAGGAGAGTTC	
EXPL2R	CATTTTGCCGTCGTAGCCTG	
WSD1-F	TGGCGAAGGGTTCAAAGTGT	
WSD1-R	AGCTTCTACTGCCTTTCCTCC	
SAUR64-F	AGTGCTACTAGCTCAACCGC	
SAUR64-R	TAGGTCCACCAGTTGGGAGG	
AT1G20823F	TGTTGCCAGGTGTCACAAGT	
AT1G35230F	CTCCCTCAGCTCCTACCACT	
AT5G52760F	CATGACCGCAAAGAACGCTG	
AT1G20823R	CTTCGCCTTGCTTGATTCGG	
AT1G35230R	GGAGAGCCACTTAGGGGAGA	
AT5G52760R	TCGAGAAACGGTGACAAGCG	
AT4G01870F	CATGTGAGTTTCAATAAAGATGGTG	
AT4G01870R	CGTCTAATTTCACAACGTACAAATC	
AT1g11545F	AATCGGGAGATGCGACATTC	
AT1g11545R	CGTGTAGCCCAATCGTCAGC	
AT3G07010F	CTAAGGAGGTGACTAAGAGAGAGTAC	
AT3G07010R	CGACGAGTGAGGATGATTTG	
AT1G22400F	GCGGATCAACGCTGGAGATA	
AT1G22400R	AACTCTCATCTTTTAGCGGACA	
AT4G18010FBH1	CGCGGATCCTTGTGAATCAAACTAATTTATTTAAGTAGC	TRANSIENT EXPRESSION
AT4G18010RHD3	CGCAAGCTTCTTCTTAGATCTCAGAAAAAGATTTTGTTTC	
AT4G00360FBH1	CGCGGATCCTCTCTTGATACAATGCATATAGAAACTGAC	
AT4G00360RHD3	CGCAAGCTTATCAATGAATATGAAATGATACTAAAATGG	
AT1G22400F	CGCGGATCCGGTGTTTCAATGGGGGTGCTC	
AT1G22400R	CCCAAGCTTCAAATGTGAGTTTTGGTTTGCCTTTG	
AT5G17860F	CGCGGATCCCGGTTACGTTGCATAAATATTTATCTG	

AT5G17860R	CCCAAGCTTGTGGAACAATAATGAGGTGTGTTAG	
AT4G14365F	CGCGGATCCGATCCACGTACGATTTTCTTAGG	
AT4G14365R	CCCAAGCTTGAAGGAATATCAAATGAATTGAACTTAG	
AT3G19720F	CGCGGATCCCACAAGAGAGACCTTTAAGAAATG	
AT3G19720R	CCCAAGCTTGTCTCTTACGAAAATGAGCAAGAG	
4G18010BDF	AAACCGAAAACACGTGTGAGAAAGAAGAAA	EMSA EXPERIMENT
4G18010BDR	TTTCTTCTTTCTCACACGTGTTTTCGGTTT	
4G18010BDMF	AAACCGAAAACATTTTTTAGAAAGAAGAAA	
4G18010BDMR	TTTCTTCTTTCTAAAAAATGTTTTCGGTTT	
4G00360BDF	ATTACCTAACTATACATGTGTAATGTGTTC	
4G00360BDR	GAACACATTACACATGTATAGTTAGGTAAT	
4G00360BDMF	ATTACCTAACTATAAAAAAATAATGTGTTC	
4G00360BDMR	GAACACATTATTTTTTTATAGTTAGGTAAT	
AT4g18010qPCR1F	GTATGGTCGGTACAATCAGTC	RD26 and BES1 ChIP and reChIP
AT4g18010qPCR1R	AACTGCAACTTGCGATCCAC	
AT4g18010qPCR2F	GGTATGTTTGGCAAATTCAGC	
AT4g18010qPCR2R	TAAGATGGTGTAGACTGTAGG	
AT4g18010qPCR3F	GTATGGTCGGTACAATCAGTC	
AT4g18010qPCR3F	AACTGCAACTTGCGATCCAC	
UBQ5F	AAGATCCAAGACAAGGAAGG	
UBQ5R	GAAGAACAGCGAGCTTAACC	
At4g18010P1 F	CGGGGTACCTTGTGAATCAAACTAATTTATTTAAGTAGC	Yeast One-hybrid assays
At4g18010P1 R	CGCGTCGACAAAGTGAGAATCCCTCACATTTAAAAG	
At4g18010P2 F	CGGGGTACCCAAAGAAAAGATATAGACAATTTTAAGACT	
At4g18010P2 R	CGCGTCGACAACTATATTTTGACAAGCAGTTTTGCG	
At4g18010P3 F	CGGGGTACCACTAATTCTCAAATAATCACTTTTACTCCG	
At4g18010P3 R	CGCGTCGACCTGCAACTTGCGATCCACT	
At4g00360P1 F	CGGGGTACCTCTCTTGATACAATGCATATAGAAACTGAC	
At4g00360P1 R	CGCGTCGACTGACTTGTGTACGCTGCAACT	
At4g00360P2 F	CGGGGTACCATTATGTACTTCTATCATATATTTATGATATTGC	
At4g00360P2 R	CGCGTCGACTGATATTTAATCTTCTCAATTTGGTTTATC	
At4g00360P3 F	CGGGGTACCAAATCCCTCACAAGAATTGAGAACTG	
At4g00360P3 R	CGCGTCGACAGCTAAATTAAATGAGATTAGTTGAGAACC	
RD26 GAD F EcoRI	CGCGAATTCATGGGTGTTAGAGAGAAAGATC	
RD26 GAD R XhoI	CCCCTCGAGTCATTGCCTAAACTCGAATGTTTGACC	
